# Prevention and Treatment of *Staphylococcus aureus* Biofilms Using Promising Agr-QS-Targeting Anti-Biofilm Agents

**DOI:** 10.3390/pathogens15080795

**Published:** 2026-07-27

**Authors:** Salma Waheed Sheikh, Ahmad Ali, Asma Ahsan, Fei Shang, Ting Xue, Lauren Gollahon

**Affiliations:** 1 Department of Biological Sciences, Texas Tech University, Lubbock, TX 79409, USA; salsheik@ttu.edu (S.W.S.); ahmaali@ttu.edu (A.A.); 2 Medical School, Twin Cities Campus, University of Minnesota, Twin Cities, Minneapolis, MN 55455, USA; ahsan046@umn.edu; 3 School of Life Sciences, Anhui Agricultural University, Hefei 230036, China; shf@ahau.edu.cn

**Keywords:** *S. aureus*, biofilm-associated infections, Agr-QS system, natural products, anti-biofilm agents

## Abstract

*Staphylococcus aureus* (*S. aureus*), a leading cause of nosocomial infections, contributes significantly to increased morbidity and mortality, especially when it forms biofilms on medical devices. This pathogen, specifically methicillin-resistant *S. aureus* (MRSA), remains a challenge to treat due to its ability to form biofilms and rapidly develop resistance against antibiotics. Biofilm formation allows bacteria to adhere to biotic and abiotic surfaces, creating a protective matrix that shields them from immune responses and antibiotic therapies. The widespread prevalence of multidrug-resistant *S. aureus* biofilms poses a significant therapeutic challenge in clinical settings. Several novel therapeutic strategies have been developed to combat *S. aureus* biofilm-associated infections. Accumulating evidence suggests that natural plants and their derivatives possess antimicrobial and chemo preventive properties that can disrupt established biofilms. Several plant-derived compounds with anti-biofilm activities have been reported to target the regulatory proteins involved in the Agr quorum sensing (Agr-QS) system, underscoring their potential as therapeutic candidates for the prevention and treatment of biofilm-associated infections. However, despite these encouraging findings, clinical validation of these plant-based agents is essential to ensure their efficacy, safety, and optimal application in treating *S. aureus* biofilm infections. The continued exploration of natural biofilm inhibitors anticipates the urgent need for new treatments to combat biofilm-associated infections and multidrug-resistant pathogens like MRSA. This review provides a detailed overview of preventive and therapeutic interventions to eradicate biofilm-forming *S. aureus* infections.

## 1. Introduction

*Staphylococcus aureus* (*S. aureus*), a Gram-positive bacterium, is a major human pathogen in both inpatient and outpatient settings and contributes significantly to morbidity and mortality [1]. About 30–50% of healthy individuals in the United States have *S. aureus*, and one in a hundred of these people is colonized with methicillin-resistant *S. aureus* (MRSA). Therefore, this antibiotic-resistant pathogen can easily be transmitted through direct contact, exposing a large population to infection [2]. In recent years, the cases of MRSA infections in hospitalized patients have reduced in several countries, but the COVID-19 pandemic had a significant impact on antimicrobial resistance, resulting in a 15% increase in nosocomial MRSA infections in the United States [3]. *S. aureus* colonizes indwelling medical devices, including catheters, implants, joints, and artificial heart valves, and produces biofilms [4,5]. The infections associated with *S. aureus* biofilms are difficult to treat, as biofilms provide a phenotypic resistance mechanism that further protects the pathogen from antibiotics and host defense [6].

The antibiotic resistance of *S. aureus* is primarily mediated through its ability to evade the host’s immune system. This is achieved through the bacterium’s ability to invade the epithelial cell linings and form recalcitrant biofilms [7,8]. Key cell wall components, such as teichoic acid polymers, and the production of virulence factors, including coagulase and toxins, play crucial roles in the adhesion and invasion processes [7,8].

Though antibiotics are the first choice to combat bacterial infections, resistance to last-resort antibiotics like vancomycin by vancomycin-resistant *S. aureus* (VRSA) has led to the emergence of multidrug-resistant strains, posing a major concern [9]. The infections caused by antibiotic-resistant *S. aureus* strains increase hospital stays and mortality, resulting in a substantial financial burden on the medical industry. Over the past decade, the total hospital costs to treat *S. aureus* infection have been estimated at $450 million [10,11]. Research on *S. aureus* biofilm formation has improved our understanding of the intricacy of *S. aureus* pathogenesis and advanced the development of therapies to prevent and treat biofilm infections. Recently, natural plants and their extracted phytochemicals have gained significant attention for their potential antimicrobial and anti-biofilm properties [12]. These green alternatives consist of complex mixtures of various compounds, making it difficult for bacteria to develop resistance to these multi-component treatments [13].

A thorough understanding of the cellular communication mechanisms within a biofilm matrix can lead to the development of new strategies and targets for identifying novel therapies against biofilm-associated *S. aureus* infections. This review outlines the mechanisms of *S. aureus* biofilm formation and highlights recent advances in the use of various natural compounds as alternative therapeutic approaches.

## 2. Prevalence of *Staphylococcus aureus*

*S. aureus* is a persistent resident of the human nasal passages and epidermal surfaces and substantially increases the risk of invasive infection. As an opportunistic pathogen, it can colonize various sites, including the skin, blood, soft tissues, lungs, bones, brain, and heart valves, causing diseases ranging from superficial skin infections to life-threatening bacteremia, endocarditis, pneumonia, and sepsis (Table 1). The two most prevalent strains, MRSA and methicillin-susceptible *S. aureus* (MSSA), are known to cause mild skin infections that can progress to life-threatening sepsis and even death [14].

**Figure 1:**
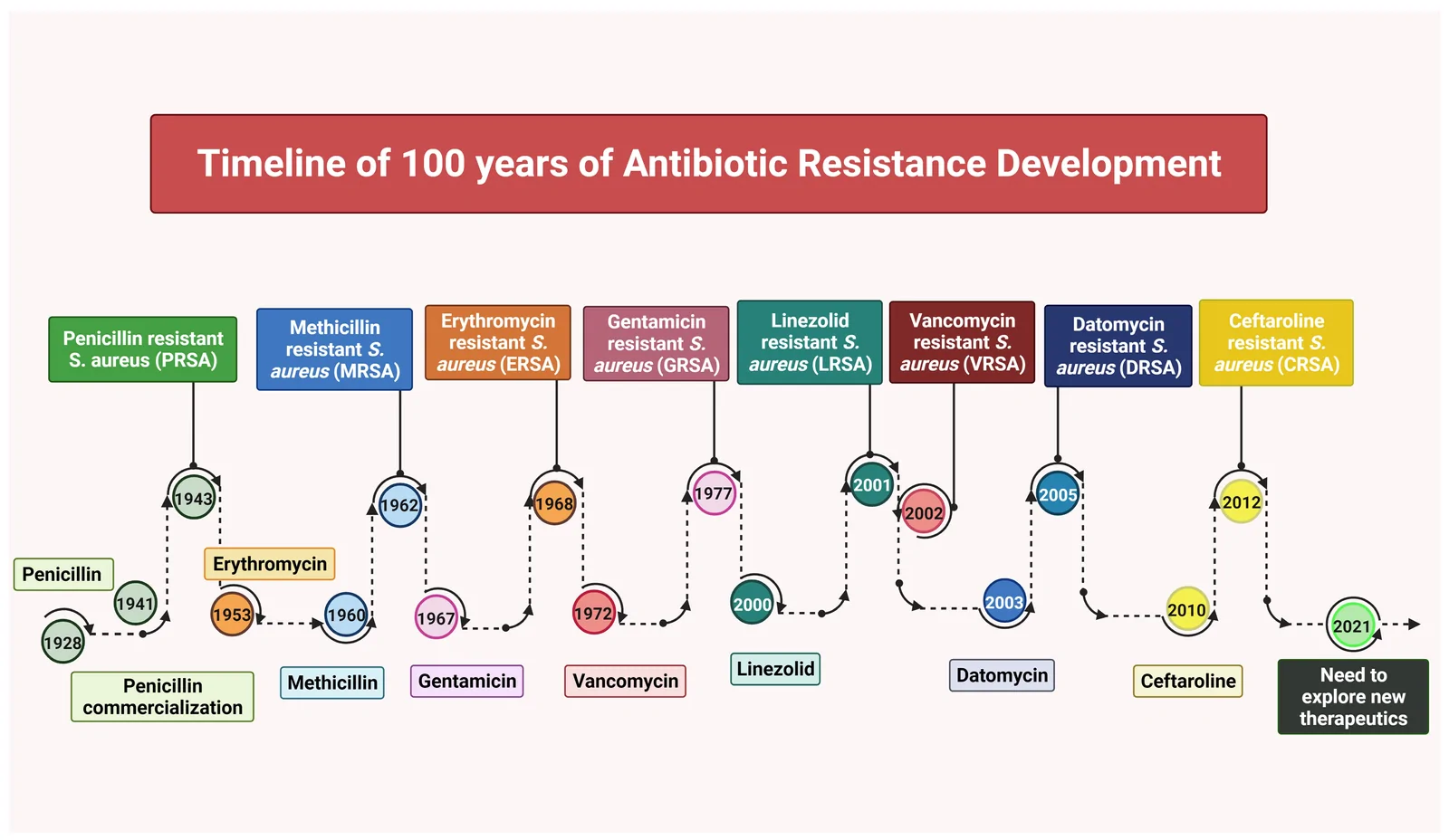
Timeline of antibiotic resistance development in *S. aureus* isolates and subsequent emergence of corresponding antibiotic-resistant *S. aureus* strains. The dashed arrows indicate the chronological progression of events, whereas the solid curved arrows indicate the transition from antibiotic introduction to the first reported resistant strain. Matching colors identify each antibiotic and its corresponding resistant phenotype. Original image created in BioRender. Gollahon, L. (2026) https://BioRender.com/dqb4rtl (accessed on 12 July 2026).

MSSA infections can be treated using beta-lactam antibiotics, whereas MRSA infections are commonly treated with sulfamethoxazole–trimethoprim, daptomycin, telavancin, clindamycin, linezolid, tigecycline, quinupristin–dalfopristin, and vancomycin [21,22]. However, the effectiveness of conventional antibiotics has substantially reduced due to increasing prevalence of antimicrobial resistance (Figure 1, Table 2), highlighting the urgent need to develop alternative therapeutics.

The clinical persistence of *S. aureus* is mostly attributed to the biofilm-mediated protection from antimicrobial agents that helps maintain chronic infection. Beyond humans, *S. aureus* is also a significant cause of mastitis in bovine, ovine, and caprine species, leading to a notable decrease in milk production and quality, resulting in substantial economic losses for the dairy industry [23]. Consequently, understanding the mechanisms regulating biofilm formation and virulence in *S. aureus* can pave a path in the development of effective strategies.

**Table 2:** Therapeutic limitations and emerging resistance associated with antibiotics used against *S. aureus*.

Infection	Antibiotic	Clinical Implications	Major Therapeutic Limitations	Emerging Resistance	References
Endocarditis	Linezolid	To treat MRSA-like cases when IV access or oral step-down is needed	Myelosuppression, especially thrombocytopenia, dose- and duration-dependent toxicity	Linezolid resistance	[24,25,26,27]
Severe community- acquired pneumonia	Daptomycin	To treat MRSA, but generally not recommended for pneumonia	Inactivated by pulmonary surfactant	Increased daptomycin resistance	[26,28,29]
Bacteremia and vertebral osteomyelitis		To treat MRSA bacteremia and deep infections	Increased CPK levels, myopathy risk, need for higher dosing in severe infection	In vivo-acquired resistance and treatment failures in MRSA bacteremia	[30,31,32]
Sepsis and septic shock	Vancomycin	Standard therapy for severe MRSA infection	Nephrotoxicity; infusion reactions (red man syndrome)	Increasing vancomycin-intermediate *S. aureus* (VISA) prevalence and rare VRSA. Recent sepsis guidelines highlighted reduced efficacy with elevated MICs	[33,34,35]
Chronic skin ulcers		Used for susceptible isolates but often limited in biofilm-rich chronic disease	Toxicity limits prolonged courses, poor penetration, and tolerance in biofilms	Increasing prevalence of VISA/VRSA along with reduced vancomycin susceptibility	[35,36,37]
Skin and soft tissue infections	Clindamycin	Commonly used for susceptible MSSA/MRSA SSTIs	Gastrointestinal toxicity kills beneficial bacteria and allows harmful *C. difficile* to grow	Substantial inducible clindamycin resistance in MRSA and MSSA	[38,39,40]
Mixed (samples taken from hospital)	Quinupristin– dalfopristin	Used for highly resistant Gram-positive infections only	Infusion-related pain, arthralgia/myalgia, limited routine use	Plasmid-mediated resistance in *S. aureus* and other staphylococci	[26,41,42]
Mixed (samples taken from hospital)	Trimethoprim–sulfamethoxazole (TMP-SMX)	Useful for selected MRSA SSTIs and some invasive infections	Hypersensitivity; hematologic toxicity	TMP–SMX resistance in MRSA and MSSA	[42,43]
Mixed (samples taken from hospital)	Tigecycline	Alternative for complicated skin/soft tissue and intra-abdominal infections	Nausea/vomiting, low serum levels unsuitable for bacteremia	Emergence of tigecycline resistance in *S. aureus* and other Gram-positive pathogens	[26]

Note: All abbreviations are listed at the end of the manuscript.

## 3. Virulence Potential of *Staphylococcus aureus*

Among all staphylococcal bacteria, *S. aureus* is the most lethal pathogen, causing frequent outbreaks over the years. This is primarily due to its wide array of virulence factors, with biofilm formation being a significant contributor to its pathogenicity (Table 3) [44]. In addition, the key feature of highly recalcitrant *S. aureus* infections is their strong adherence and invasion capabilities [45,46]. The rise in multidrug-resistant strains, along with the presence of antibiotic residues in food products such as dairy and meat, further intensifies the challenge.

## 4. Biofilm Formation by *Staphylococcus aureus*

### Biofilm Development—Attachment, Proliferation, and Detachment

**Figure 2:**
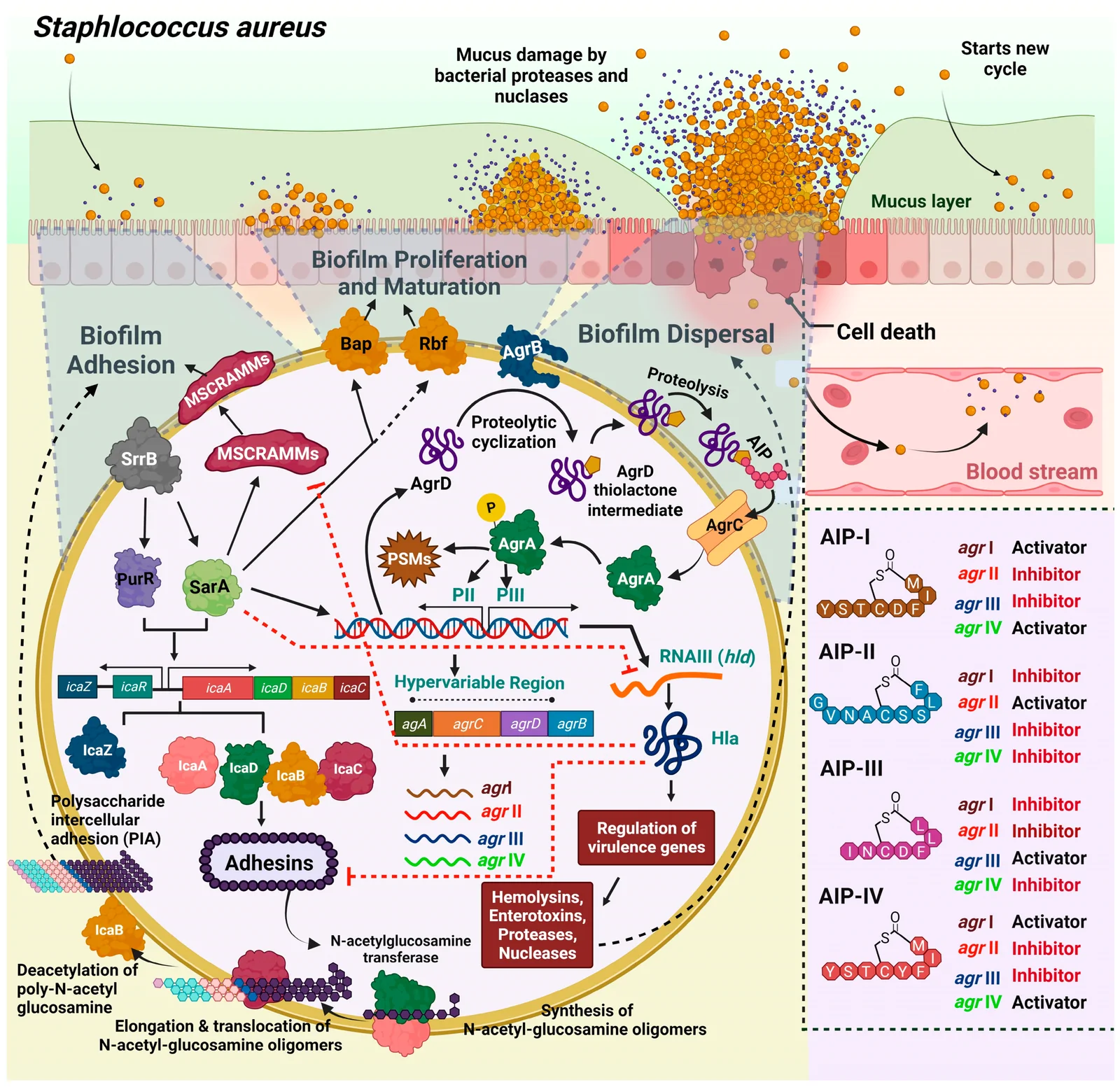
Mechanistic regulation of biofilm attachment, proliferation, maturation, and dispersal in *S. aureus*. AIP, auto-inducing peptide; MSCRAMMs, microbial surface components recognizing adhesive matrix molecules; PSMs, phenol soluble modulins. Original image created in BioRender. Gollahon, L. (2026) https://BioRender.com/dqb4rtl (accessed on 12 July 2026).

Biofilms are complex, structured communities formed by sessile microbes, allowing the bacteria to adhere and synthesize an organic matrix. The development of *S. aureus* biofilm can be divided into three phases: bacterial attachment to the host, proliferation leading to a mature biofilm formation, and detachment to initiate a new biofilm cycle (Figure 2). During initial attachment, MSCRAMM adhesions aid in host tissue colonization [47,48,57]. The LPXTG motifs in MSCRAMM adhesins form strong covalent bonds with various human matrix proteins, initiating biofilm formation at the infection site [58]. Other surface proteins, including Sdr proteins (Serin-aspartate repeat family), autolysins (Atl), and accumulation-associated proteins (Aap), are also involved during the initial stages of biofilm formation [57,59]. During maturation, the synthesis and excretion of polysaccharide intercellular adhesins (PIAs/PNAG), regulated by the *icaADBC* operon-coded enzyme, provide adhesion between the cells (Figure 2) [49,50,60]. PIA plays a vital role in determining the structure of the growing biofilm during the infection. Its expression is significantly upregulated by the *sarA* and *sigB* genes; *luxS* negatively regulates the expression of PIA and associated genes [60]. The expression of the *ica* operon is regulated by the activation of SarA and Pur proteins. N-acetyl-glucosaminyl transferase, a transmembrane protein produced by the expression of *icaAD* genes, is required to produce N-acetyl-glucosamine oligomers [52]. Additionally, the co-expressed *IcaC* gene is responsible for the elongation and translocation of the growing polysaccharide to the cell surface. Finally, the deacetylation of this poly-N-acetylglucosamine occurs through the action of a surface protein, IcaB, contributing to PIA development. However, PIA is not present in all *S. aureus* isolates. Other adhesion proteins, such as Aap (accumulation-associated protein), fibrinogen-binding proteins (FnbpA and FnbpB), protein A, extracellular matrix-binding protein (Embp), and surface protein G (SasG), are involved in biofilm formation and maturation [60,61].

In addition to PIA/PNAG, many clinical *S. aureus* isolates form biofilms whose matrices are predominantly proteinaceous or eDNA-rich rather than polysaccharide [62,63]. Protein-dependent biofilms are mediated by surface adhesins and secreted proteins, including SasG, protein A, fibronectin-binding proteins (FnBPA/FnBPB), clumping factor B, serine–aspartate repeat proteins, and biofilm-associated protein (Bap), which can drive robust biofilm formation even in *ica*-negative or PIA-independent strains [64,65,66]. Recent genetic and biochemical studies further showed that secondary messenger signaling and global regulators also contribute to these non-PIA biofilms. For example, the changes in the secondary messenger cyclic-di-AMP (mediated by the phosphodiesterase GdpP) and regulators like SarA and SigB determine how much eDNA is released and how the matrix is assembled, underscoring that PIA-independent, protein/eDNA-dominated biofilms are common and clinically relevant in *S. aureus* [50,67].

Biofilm-associated protein (Bap) promotes biofilm formation and proliferation, even in the absence of exopolysaccharides. Bap was first discovered in the *S. aureus* strain responsible for bovine mastitis [68]. The expression of both the *ica* operon and the *bap* gene significantly enhances the biofilm formation and invasion capabilities of *S. aureus* isolates. In clinical *S. aureus* strains, increased expression of the *rbf* gene activates the *ica* operon. However, the *rbf* gene has not been observed in any bovine *S. aureus* isolates [69].

Detachment, the final step in biofilm formation, involves the disruption of non-covalent interactions between biofilm cells and the supportive matrix [53].

## 5. Biofilm Development and Its Relation to Quorum Sensing

Within the biofilm matrix, bacteria use certain chemical signaling molecules to communicate with one another, a process known as quorum sensing (QS). The QS system plays an intriguing role in the development of biofilm resistance against different antimicrobial agents [50]. The QS system allows the bacteria to sense and respond to the accumulation of various signaling auto-inducing peptides (AIPs) secreted within the matrix [70,71]. During biofilm maturation and dispersion, the kinase receptors on the bacterial surface bind to high concentrations of these AIPs in the matrix and transmit signals to trigger the expression of *sarA* and an accessory gene regulator, Agr [53]. The *S. aureus* Agr system is essential for the production of several virulence factors, including toxins and degradative exoenzymes [56].

### 5.1. Role of Agr System in Biofilm Dispersal

In *S. aureus* isolates, the biofilm maturation and dispersal processes are tightly regulated by the Agr-QS system. Repression of *agr* operon genes contributes to biofilm formation, while activation of the *agr* system triggers the detachment of mature biofilms [53]. The Agr system is a highly integrated signaling network that regulates virulence by sensing the concentration of signaling molecules and bacterial cell density in the matrix [55]. The *agr* operon comprises two divergent promoters, P2 and P3, which drive the transcription of two distinct RNA molecules, RNAII and RNAIII (Figure 2) [54]. RNAII is a polycistronic mRNA that codes for four Agr proteins, including AgrC, AgrA, AgrB, and AgrD. A histidine kinase, AgrC, along with response regulator AgrA, forms a two-component signal transduction system [55]. AgrB is a multifunctional protein that functions both as an endopeptidase and a chaperone, aiding in the maturation and export of AIPs. AgrD serves as a precursor of the AIP pheromone, which is proteolytically processed by AgrB to form a thiolactone intermediate, as shown in Figure 2. This intermediate is subsequently exported across the membrane, where it is cleaved to generate a mature AIP pheromone. *S. aureus* strains can be divided into four different groups based on the polymorphic variant produced by the *arg* locus: *agr*I, *agr*II, *agr*III, and *agr*IV (Figure 2) [72]. Species-specific variants have been widely reported; some studies have also reported the association of *agr* variants with different *S. aureus* features (Table 4). Notably, isolates with the *agr*I variant exhibit an increased tendency to invade MAC-T cells, while the isolates with *agr*II showed a greater reliance on biofilm formation [73].

When the level of mature AIP reaches its threshold, it activates AgrC. Upon activation, AgrC initiates a phospho-relay cascade that results in AgrA phosphorylation. Phosphorylated AgrA binds to the P2 promoter, and upregulates the transcription of RNAII (positive feedback mechanism) and the P3 operon. The P3 operon, which is an AgrA-dependent operon located adjacent to P2, encodes RNAIII (*hld*), a posttranscriptional regulator that controls the expression of multiple virulence factors, including proteases, nucleases, the biofilm dispersal gene (*hla*), toxins, and surfactants. This ultimately leads to biofilm dispersal as depicted in Figure 2 [74]. Activated AgrA also regulates the *psm-mec* gene in an RNAIII-independent manner, leading to the production of phenol-soluble modulins (PSMs). PSMs such as δ-hemolysin, PSMα1-4, PSMmec, and PSMβ1-2 contribute to the degradation of exopolysaccharides (EPSs), facilitating biofilm dispersal and promoting host cell perturbations. During the dispersal phase, mature biofilm ruptures, releasing bacterial aggregates that can seed the formation of new biofilms [48,61].

**Table 4:** Characteristic features of different Agr variants and their role in biofilm regulation.

Agr Variant	Characteristic Features	Biofilm Regulation	Reference
*agr*I	Predominantly reported in CA-MRSA and methicillin-resistant bovine isolates	Regulates a broad set of toxins, proteases, and adhesins; *agr*I dysfunction is linked to prolonged bacteremia and altered biofilm behavior.	[75,76]
	High prevalence of resistance towards beta-lactamase, glycopeptides, fluoroquinolones, aminoglycosides, tetracyclines, macrolides, lincomycins, and sulphonamides		[77]
*agr*II	Predominantly causes nosocomial MRSA and MRSA bloodstream infections	*agr*II activity influences biofilm maturation and dispersal. It is associated with distinct toxin/adhesin profiles.	[54,75]
	Toxic shock syndrome		
	Prolific biofilm producers		[78]
*agr*III	Predominantly causes CA-MRSA	Linked with toxin-mediated virulence, *agr*III mutants show altered biofilm and persistence phenotypes.	[75,79]
	Potent biofilm producers		[80]
*agr*IV	Predominantly reported in swine farm isolates and generalized exfoliative syndromes	Regulates virulence and biofilm, but detailed functional data are limited.	[54,77]
	High prevalence of resistance to fluoroquinolones, aminoglycosides, tetracyclines, macrolides, lincomycins, and sulphonamides		[77]
	High prevalence of enterotoxin genes		[54]

Note: All abbreviations are listed at the end of the manuscript.

### 5.2. Alternative Anti-Biofilm Strategies

The global rise in MRSA and VRSA is significantly undermining the effectiveness of nearly all reported antibiotics [81]. Unfortunately, no vaccines have been approved to treat *S. aureus* infections. In addition, recalcitrant *S. aureus* biofilms require surgical debridement and prolonged antimicrobial therapy. The global market for antibiotics to treat *S. aureus* infections, especially MRSA, was valued at 984.6 million US dollars in the year 2020, with a projected Compound Annual Growth Rate (CAGR) of 4.4%, expected to reach approximately 1327.9 million US dollars by 2027 [82]. Consequently, various natural and synthetic anti-biofilm agents are being explored as an alternative to conventional antibiotics. These include the metabolites extracted from other prokaryotes to target the Agr-QS system or to degrade the biofilm matrix (Table 5), as well as “green synthesized” nanoparticles and bacteriophages to combat *S. aureus* biofilms [83,84].

### 5.3. Green Alternatives as Potential Medicinal Therapeutic Agents

To combat this growing threat, it is crucial to explore unconventional therapies with antimicrobial and anti-biofilm properties. While approaches like bacteriophages, synthetic compounds, and nanotechnologies hold potential, they often face limitations such as cytotoxicity and high cost (Table 5). This dire situation urges us to reconsider “traditional” green alternatives, though for most biofilm applications the current reports are largely limited to in vitro and early preclinical studies. Plants, for instance, serve as a huge reservoir for secondary metabolites (phytochemicals) with known medicinal properties. Over 80% of commercialized medicines are derived directly or indirectly from natural sources, with more than 50% containing active compounds isolated from plants, herbs, and minerals [13]. These compounds play an important role in modern healthcare. However, some remedies still require experimental validation, and clinical trials are needed to commercialize their use.

Plants produce two types of active metabolites: primary and secondary metabolites. Primary metabolites are the building blocks of an organism undergoing various metabolic processes to produce secondary metabolites. Secondary metabolites assist them in surviving and reproducing [129]. These metabolites play a crucial role in chemical defense, protecting plants from abiotic stress while also contributing to floral scents and pigments. The most indispensable benefit, however, is their role as a source of medicines and industrial additives. Based on their biosynthetic pathways, secondary metabolites can be classified into several categories, including alkaloids, glycosides, steroids, phenols, terpenoids, and tannins. These compounds exhibit biodynamic medicinal properties, applicable to both human and animal health [129,130].

Phenols and polyphenols are the most potent compounds, extensively reported for their antimicrobial activities. They have a unique ability to bind to a wide range of proteins and glycoproteins and are often used to enhance the antimicrobial properties of antibiotics [131,132]. Tannins, which are polymeric phenolic compounds, have demonstrated antibacterial and anti-biofilm-forming properties due to their ability to inhibit enzymes involved in adhesion and transport [133,134,135]. Flavonoids, the most diverse group of metabolites, are well-known for their antibacterial effects. Based on their chemical structure, they are classified as flavones, isoflavones, flavonols, flavanonols, flavanones, chalcones, and anthocyanides. Flavonoids are known to induce oxidative stress and block the transport of electrons during respiration [136]. Quinones, particularly anthraquinones, are another class of highly reactive compounds that can form irreversible complexes with bacterial adhesins, leading to biofilm dysfunction [132]. Plants also produce terpenes and terpenoids, which interact with other species. Most essential oils, rich in terpenoids, exhibit both antibacterial and anti-biofilm activities [137,138].

**Figure 3:**
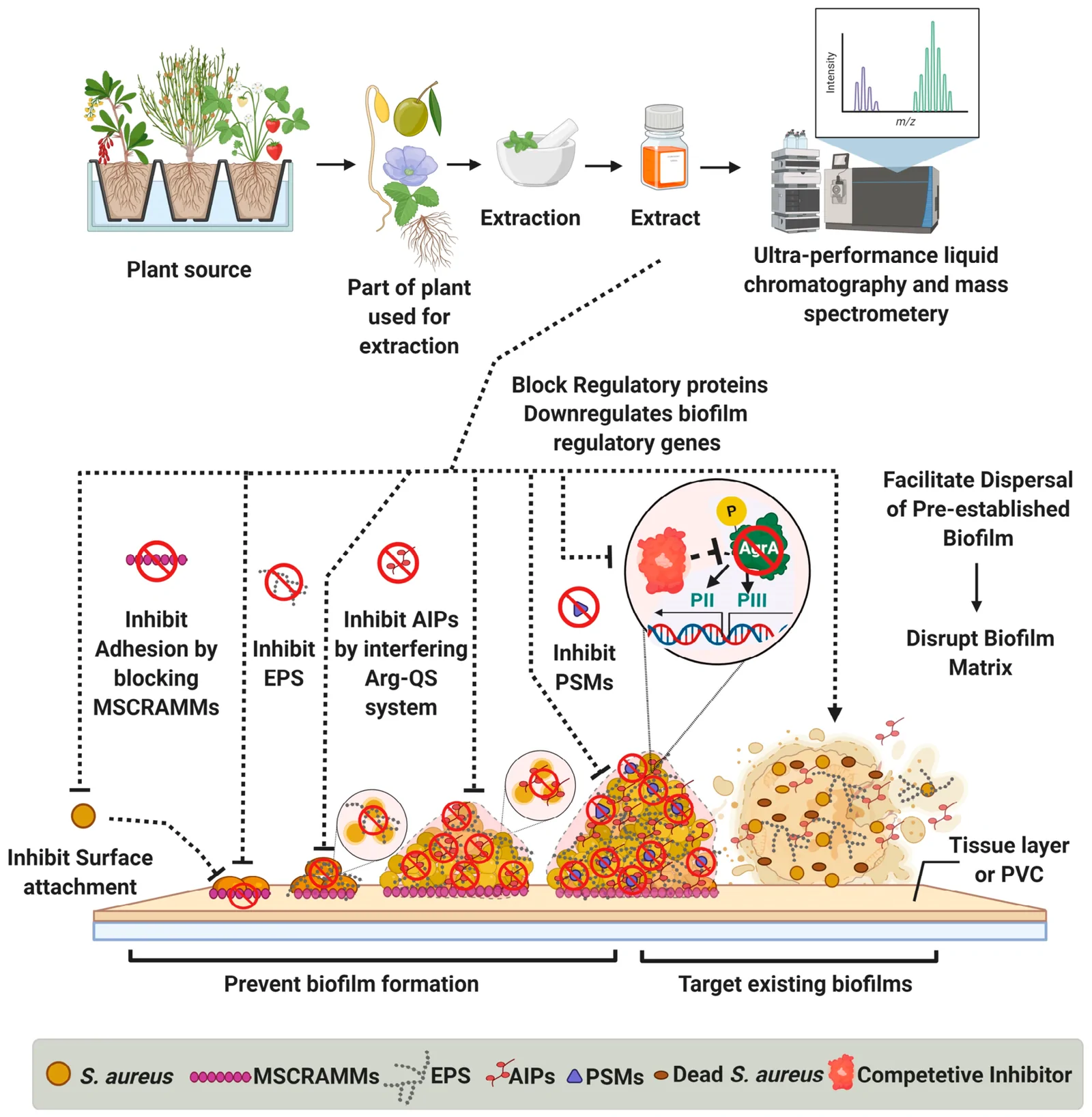
Schematic Representation of plant-based anti-biofilm agents that target attachment, adhesion, polymer matrix, or QS System to inhibit biofilm formation and facilitate dispersal of pre-established biofilms. Original image created in BioRender. Gollahon, L. (2026) https://BioRender.com/dqb4rtl (accessed on 12 July 2026).

Alkaloids are well-known nitrogen-containing natural bioactive compounds that are a rich source for drug discovery. These compounds inhibit bacterial growth by interfering with DNA intercalation and suppress bacterial proliferation and accumulation during biofilm formation [131,132,136]. Numerous alkaloids extracted from medicinal plants and herbs contribute to several biological and pharmacological uses. Compared to commonly used antibiotics, alkaloids provide enhanced resistance, which has led to cutting-edge research aimed at exploring novel therapeutic approaches [132,136]. Phytochemicals represent a complex reservoir of active compounds with a broad though often vague spectrum of activity. Many plant-based anti-biofilm targets mainly interfere with adhesion, attachment, formation of a polymer matrix, or blocking the QS system, which ultimately leads to biofilm inhibition (Figure 3).

Numerous medicinal plants and plant-derived phytochemicals have been reported to inhibit *S. aureus* biofilm formation. This protection is achieved by inhibiting initial bacterial adhesion, membrane disruption, and modulating the regulatory genes (*agr*, *sarA*, and *ica*) [52,53]. Table 6 summarizes major findings of the most reported plants, including major bioactive constituents, proposed targets, anti-biofilm mechanisms, experimental models, and developmental stage.

### 5.4. Camellia sinensis

*Camellia sinensis* (*C. sinensis*) is a flowering plant of the *Theaceae* family with evergreen shrubs whose leaves and leaf buds are used to make green tea. This tea plant is a hub of beneficial theophylline, flavonoids, saponins, and several polyphenol derivatives including epicatechin (EC), epigallocatechin (EGC), epicatechin gallate (ECG), and epigallocatechin gallate (EGCG) [165,166]. Tea leaf extract is reported to inhibit the growth of various bacterial species, including *S. aureus*, and possesses antimicrobial, antiviral, and anti-biofilm properties [166,167]. Green tea extract contains a putative anti-adhesive component that inhibits bacterial attachment and development of biofilms, and disperses pre-existing biofilms at concentrations even lower than MIC, suggesting the potential for pharmaceutical applications [140,141,168,169].

### 5.5. Moringa oleifera

*Moringa oleifera* (*M. oleifera*), commonly known as drumstick or Miracle tree, is a fast-growing Moringaceae family tree particularly valued for its rich nutritional content, including fatty acids, carotenoids, minerals, and vitamins [170]. *M. oleifera* has been shown to possess significant antimicrobial and anti-biofilm properties, especially against *S. aureus*. Leaves and seeds of *M. oleifera* are reported to inhibit biofilm formation, with an inhibition rate of up to 99% [171,172]. Further research, particularly in animal models, is needed to better understand its full therapeutic potential.

### 5.6. Rosmarinus officinalis

*Rosmarinus officinalis* (rosemary) is a well-known medicinal plant. Experimental studies have demonstrated that methanol extracts of rosemary effectively inhibit *S. aureus* biofilm formation, with significant reductions in the minimum inhibitory concentration (MIC) values, indicating its potential as an antimicrobial agent [173,174,175,176]. Recent studies using NMR and MS fragmentation identified bioactive metabolites from rosemary extracts, such as *micromeric acid* and *oleanolic acid.* These compounds demonstrated complete inhibition of MRSA biofilm formation, supporting the plant’s potential as a treatment for biofilm-related infections [177].

### 5.7. Psidium guajava

*Psidium guajava* is a fruiting plant known for its broad range of therapeutic activities, including antimicrobial effects [178,179,180,181]. Several studies have reported that the methanol extract of *P. guajava* leaves inhibits biofilm formation by downregulating the expression of critical biofilm formation genes such as *icaAD*, *sarA*, and *agr* [151,182]. Ultra-performance liquid chromatography (UPLC) and GC-MS analyses identified compounds like L-5-Propylthiomethylhydantoin and several phenolics that inhibit biofilm formation without causing cytotoxicity. Therefore, their pharmaceutical use may be a justification for clinical trials [182,183].

### 5.8. Eucalyptus

Eucalyptus is particularly known for its medicinal, commercial, and ornamental applications [184]. *Eucalyptus globulus* (*E. globulus*) is a prominent evergreen tree whose leaves are used to produce essential oils with antimicrobial, antioxidant, and anti-inflammatory properties [185,186,187]. Studies have shown that the essential oil, aqueous and methanolic leaf extracts of *E. globulus* significantly inhibit the initial attachment and adhesion of *S. aureus* and lead to decreased virulence and biofilm inhibition [130,187,188]. The *Eucalyptus sideroxylon* (*E. sideroxylon*) species is well-known for antibacterial and anti-fungal properties [156,189]. It has been reported that the flower extract of *E. sideroxylon* possesses potent anti-biofilm activity against *S. aureus* isolates at sublethal doses (95.9% inhibition) in a dose-dependent manner [156,190].

### 5.9. Azadirachta indica

*Azadirachta indica* (*A. indica*) is a well-known medicinal plant that has been extensively studied for its antimicrobial and anti-biofilm properties against *S. aureus* [178,179,191]. Crude extracts of *A. indica* showed a 60–70% reduction in MRSA and MSSA biofilm formation and up to 80% when used in combination with *Ocimum sanctum* [192,193]. These studies underline the need for further research and clinical trials to explore the full therapeutic potential of *A. indica*.

### 5.10. Curcuma longa

*Curcuma longa* (*C. longa*) is widely recognized for its broad medicinal applications [194,195,196]. Recent research has highlighted significant antibacterial and anti-biofilm properties of *C. longa* rhizome extracts (containing curcumin) against biofilm-producing *S. aureus* isolates [168]. MRSA isolates were completely eradicated at 20 μM curcumin combined with light, with lower doses also significantly reducing survival rates, highlighting curcumin’s potential in photodynamic therapy [197,198]. These findings suggest the fact that *C. longa* derivatives can be used as potential anti-biofilm agents against *S. aureus* infections.

### 5.11. Sanguisorba officinalis

*Sanguisorba officinalis* (*S. officinalis*) is commonly known in traditional Chinese medicine for treating wounds and stopping bleeding [199]. The root extract of *S. officinalis* is rich in triterpenoid saponins and tannins, compounds known for significantly inhibiting the growth of Gram-positive bacteria as compared to Gram-negative bacteria [200,201,202]. Further studies identified that the reduction in biofilm-forming ability is due to the interaction of triterpenoid saponins with the *ica* locus and *agr* system genes [163,203,204].

### 5.12. Others

Natural products have been one of the most important sources of novel antimicrobial agents for the past decade because of their structural diversity [205]. Recent advances in isolation and separation technologies have led to the discovery of various novel metabolites that can serve as potential candidates to cure persistent bacterial infections all over the world. The screening of natural anti-biofilm agents has consistently widened. In addition to the above-mentioned anti-biofilm agents, several other plants are also reported for their role in the inhibition of biofilms, including *Allium sativum* [206], *Annona senegalensis* [207], *Jatropha curcas* L. [208], *Orostachys japonicus* [209], *Moringa stenopetala* [205,210], *Spondias purpurea* [211], *Juglans regia* L. [212], *Citrus sinensis* [213] and several others enlisted in Table 7.

Most of the above-conducted experiments are pilot studies to evaluate the effectiveness of these anti-biofilm agents against infectious *S. aureus* isolates. Furthermore, most of these anti-biofilm agents usually alter the metabolic pathways involved in biofilm adhesion and proliferation. Some studies have also identified potential agents that can disperse the pre-established biofilms on biotic and abiotic surfaces by inhibiting the *agr*-QS system and related proteins. However, the molecular mechanisms underlying the inhibition of biofilm formation and proliferation need further experimental validation and clinical trials.

### 5.13. Translational Challenges in the Clinical Development of Natural Anti-Biofilm Agents

Despite the potential in vitro activity, plant-derived anti-biofilm agents have several translational challenges that limit their progression toward clinical use [284]. This translational gap reflects a series of interconnected challenges other than effectiveness of antibiotics. In addition to being a potent biofilm inhibiting agent, these phytochemicals require several standardization and quality control checks to be marked. The lack of phytochemical standardization is the major limitation in clinical development. The chemical composition of these plant extracts can vary depending on the cultivar, species, harvesting season, extraction method, storage conditions, and geographic origin, thereby reducing reproducibility across studies [285]. This variability resulted in inconsistent batch-to-batch activity and variability in dosage and therapeutic efficiency across different studies [286]. Furthermore, most studies rely on crude plant extracts rather than chemically characterized active constituents, which further limits reproducibility and regulatory approval.

Limited understanding of the pharmacokinetic properties of many phytochemicals is another major constraint [285]. Several plant-derived compounds have suboptimal pharmacokinetic properties, including limited aqueous solubility, poor intestinal absorption, rapid metabolism, low systemic bioavailability, and inadequate tissue penetration, thereby limiting their in vivo efficacy [284]. The reported studies do not reflect these physiological characteristics accurately leading to the reduced translation of in vitro findings.

The toxicity and safety evaluation of phytochemicals also remains understudied specifically regarding long-term exposure, high dose administration, and use of combination therapies for systemic treatment [284,287]. The stability of plant-derived phytochemicals is another hurdle, as several bioactive compounds are prone to degradation when exposed to light, temperature, pH, or oxidation, thereby reducing their bioactivity and stability during administration and storage [286]. To overcome these challenges, advanced delivery approaches such as nano formulations, encapsulation, and surface-targeted delivery systems may improve the stability and bioavailability of these phytochemicals. Although these approaches seem promising, they require rigorous safety, toxicological, and pharmacological validations before clinical use [288].

The lack of well-designed clinical trials remains one of the most significant challenges, because most available evidence for plant-derived anti-biofilm agents is still limited to in vitro studies or early preclinical models [289]. Consequently, future development should prioritize standardized extract characterization, identification of active principles, pharmacokinetic and toxicological profiling, optimized formulation, and controlled clinical evaluation for realistic therapeutic options.

## 6. Conclusions and Future Prospects

*S. aureus* is included in the World Health Organization’s list of antibiotic-resistant priority pathogens that pose a hazard to human health. Biofilm production is one of the most effective survival strategies used by *S. aureus* and is extremely difficult to treat using conventional antibiotics. *S. aureus* biofilm infections are usually managed by either removing the lesions or by administering high doses of systemic/topical antibiotics, necessitating repetitive hospitalization and multiple surgical operations, which ultimately increases the cost and risks of treatment. These challenges highlight the critical importance of finding alternative therapeutics to treat and prevent staphylococcal biofilms. The identification of effective bioactive drugs provides a feasible and cost-effective approach to address the overwhelming threat of antibiotic resistance. Several innovative therapeutic strategies have been proposed to combat these infections, but only a handful have been tested in clinical trials. Most anti-biofilm agents reported in vitro require additional in vivo validation because many of these compounds have significant disadvantages and safety concerns.

Quorum sensing, specifically through the *agr* system in *S. aureus*, operates as a genetic “see-saw”. This *agr* activation triggers virulence toxins (e.g., hemolysins, proteases) for tissue invasion and these proteases also facilitate biofilm dispersal. The downstream effector RNAIII acts as a master regulator of the Agr system by shifting the bacteria from a biofilm-forming state to a tissue-invasive state. Inhibiting the *agr* system blocks the production of these matrix-degrading enzymes, allowing the biofilm to become thicker, denser, and more resistant to mechanical or chemical clearance. This trade-off often creates a dilemma in clinical settings; while anti-virulence therapies reduce acute toxicity, they can inadvertently promote long-term bacterial persistence. Most of the phytochemicals discussed in this study, including quercetin, ferulic acid, carvacrol, curcumin, tea polyphenols, and salicylic acid derivatives, act directly on the Agr-QS system. These phytochemicals have been shown to downregulate *agr* and *RNAIII* expression, resulting in reduced virulence and biofilm formation in preclinical studies. However, disruption of the Agr-locus has been reported to enhance adherence, increase biofilm formation, bacterial persistence, and adaptation to chronic infection. Clinical and experimental studies show that isolates with *agr*-dysfunction form thicker and robust biofilms, indicating that indiscriminate inhibition of Agr system could be detrimental during chronic stage infections. The therapeutic outcome is highly dependent on the host, infection site, and stage of infection. Recent in vivo studies reported that Agr primarily contributes to virulence rather than biofilm formation, highlighting the complexity of targeting this regulatory system. Nevertheless, phytochemicals that modulate Agr-QS signaling not only attenuate virulence but also disrupt biofilms, providing a more balanced therapeutic strategy to combat *S. aureus* infections and requiring further validation. Several in vitro studies reported inhibition of both MRSA and MSSA infections in response to treatment with these bioactive derivatives. Additionally, they can also synergistically work with several antibiotics or photodynamic therapies to enhance biofilm disruption in experimental models. Although inhibiting *agr* alone can promote biofilm stabilization, phytochemicals in combination with traditional antibiotics have been reported with increasing frequency to be effective against treating *S. aureus* biofilms. By pairing a QS inhibitor with an antibiotic, clinicians aim to achieve virulence attenuation alongside the clearance of persistent, non-dispersing biofilm cells.

Despite substantial progress, several limitations impede the clinical translation and therapeutic applications of plant-derived anti-biofilm compounds. The clinical application of many phytochemicals is restricted due to unfavorable pharmacokinetic properties such as low oral bioavailability, poor aqueous solubility, rapid metabolism, and limited stability under physiological conditions. Moreover, the effective in vitro concentrations required to inhibit biofilm formation may not be achievable in vivo due to potential cytotoxicity. In addition, poor standardization, batch-to-batch variability, and the lack of pharmacokinetic and toxicological studies of plant extracts pose significant challenges to clinical development. Consequently, optimization of active compounds, improved drug delivery systems, and rigorous preclinical validations are needed before these agents can be used as viable therapeutic candidates to prevent and treat *S. aureus* biofilm-associated infections.

## Figures and Tables

**Table 1:** Selected clinical and epidemiological contexts in which *S. aureus* infections and biofilms are implicated.

Clinical Epidemiology	Population	Metric Reported	Approximate Affected Population	Biofilm Resistance	References
Staphylococcal food poisoning (SFP)	Outbreak of *S. aureus* enterotoxin-mediated foodborne disease	Individuals exposed to these specific outbreaks	Up to ~85% of exposed persons	Consumption of enterotoxin-producing *S. aureus*-contaminated food and contact surfaces contributes to persistence biofilms	[15,16]
Sepsis (all causes)	Global population	Annual sepsis cases and deaths, proportion of all global deaths	~48.9 million cases and ~11 million deaths in 2017, ~20% of all deaths worldwide	MRSA leads to sepsis and bloodstream infection. It forms biofilms on intravascular devices and endovascular tissues	[17,18]
Healthcare-associated MRSA infections	Hospitalized patients	Proportion of healthcare-associated *S. aureus* infections due to MRSA	Commonly 20–50% depending on region	MRSA causes device-related and surgical site infections, frequently associated with biofilm and multidrug resistance	[19,20]
Community-associated MRSA skin and soft tissue infections	Patients with SSTIs in community settings	Proportion of purulent SSTIs caused by MRSA	Often >50% of purulent SSTIs (high variability reported)	MRSA SSTIs frequently involve biofilm on skin and soft tissue surfaces, complicating treatment and recurrence	[20,21]
Prosthetic joint and device-related infections	Patients with orthopedic implants and intravascular catheters	Proportion of device-related infections due to *S. aureus*	*S. aureus* commonly accounts for 20–40% of prosthetic joint infections and many catheter-related bloodstream infections	Biofilm formation on prosthetic materials and catheters is central to chronic, relapsing infection and antibiotic tolerance	[20]

Note: All abbreviations are listed at the end of the manuscript.

**Table 3:** Virulence factors responsible for recalcitrant *S. aureus* biofilm infections.

Virulence Factors	Associated Genes	Function	Clinical Symptoms	References
MSCRAMMs	*clfA*, *clfB*, *fnbA*, *fnbB*, *cna*, *sdr*, *bbp*	Adhesion with host tissues	Endocarditis, osteomyelitis, endoprosthesis	[47,48]
Biofilm	Locus *ica*, *arg* system	Persistence in the host	Chronic infections	[49,50]
Leukocidins (e.g., PVL and toxin γ)	*luks*-PV, *lukF*-PV	Deceive the host immune response	Invasive skin infections, pneumonia, abscesses	[51]
Capsular polysaccharides, protein A, extracellular matrix binding protein	*hlg*, *cap5*, *cap8*, *spa*, *eap*	Deceive the host immune response	Invasive skin infections, pneumonia, abscesses	[52,53]
RNAIII-dependent proteases, lipases, and nucleases	V8, *hysA*, *hla*, *plc*, *sepA*	Penetration into host tissue	Tissue lesions	[54,55]
Toxins	TSST-1, ETs, and enterotoxins	Biofilm dispersal and virulence	MTSS, NMTSS, SSSS, Bullous impetigo, SFD	[56]

Note: All abbreviations are listed at the end of the manuscript.

**Table 5:** Alternative anti-biofilm strategies and their respective limitations.

Category	Source	Targeted Action	Limitations	Level of Evidence	Reference
Synthetic Compounds	RNAIII-inhibiting protein and its derivatives	Inhibit the expression of *agr* and biofilm-producing genes	High production cost, limited sustainability	In vitro and animal models	[85,86]
	Savirin	Inhibits auto-induction and quorum sensing		In vitro and animal models	[87,88,89]
Probiotics	*Lactobacillus casei*, *Lactococcus lactis* V7, *Lactobacillus rhamnosus* ATCC 7469	Inhibit adhesion, invasion, and biofilm formation	Side effects on beneficial bacteria of the host and emergence of antimicrobial resistance. Variable colonization efficiency; delivery challenges	In vitro and animal models	[90,91,92]
Bacterial	*Streptomyces* sp. N174	Antimicrobial and anti-biofilm properties	Survival of live cells during the gastrointestinal transit and their effective delivery to target tissues. Cross-species variability	In vitro	[93]
	*Staphylococcus schleiferi*	Inhibits Agr expression		In vitro	[94]
	Marine bacteria	Competitive inhibitor of AgrC		In vitro	[95]
	3-oxo-C12-HSL, (HQNO) from *Pseudomonas aeruginosa*	Inhibits auto-induction of AIPs		In vitro	
Bacteriophages	Isolated from farmyard slurry, host: *S. aureus* DPC5246	Inhibits biofilm proliferation	Bacterial resistance, immunogenicity, co-evolutionary dynamics, narrow host range, and difficulty in phage delivery to the target site. Host specificity	In vitro	[96]
	ᶲ SA012			In vitro	[97]
	Polyvalent phage K	Inhibits biofilm formation		In vitro and animal models	[98,99]
	Bacteriophage K and DRA88	Reduced biofilm		In vitro	[100]
Snake venom lectins	*Bothrops jararacussu*	Biofilm disruption	Small-size peptide hinders its large-scale production and toxicity evaluation	In vitro	[101]
Nanoparticles	Silver and gold	Anti-biofilm properties	Expensive production, high doses exert cytotoxic and genotoxic effects	In vitro	[83,84]
	Silver and Zinc oxide with nitric oxide	Inhibit biofilm formation		In vitro	[102]
	Silver and Zinc oxide nanoparticles in combination with Antibiotics	Dispersion of biofilm		In vitro	[103]
	Phosphatidylcholine-decorated gentamicin-loaded gold nanoparticles	Anti-biofilm		In vitro and animal models	[104,105]
	Magnesium fluoride and yattrium fluoride nanomaterials	Reduce colonization		In vitro	[106,107]
Hormones	17β-Estradiol	Invasion	High cost, less sustainability	In vitro	[108]
AIP and AIP derivatives	Truncated AIP-I, II, III,	Inhibits auto-induction of AIPs	High cost	In vitro and animal models	[59,109]
Fungal	12 compounds from marine-derived fungi	Inhibit biofilm formation	High cost, less sustainability	In vitro	[110]
Enzymes	Lysostaphin	Disrupts biofilms	Poor retention, enzymatic stability, and activation of immune responses	In vitro, animal models, and early clinical evaluation	[111,112,113,114]
	Cysteine histidine-dependent amidohydrolase/peptidase	Disrupts biofilms		In vitro	[115]
	Endolysins	Disrupt biofilms		In vitro and animal models	[116,117,118]
	V8 protease	Inhibits biofilm formation and promotes biofilm detachment		In vitro	[119]
	Staphopains	Affects biofilm integrity		In vitro	[119,120]
	Aureolysin	Inhibits biofilm formation and disperses preformed biofilms		In vitro	[121]
	Cysteine proteases	Anti-biofilm activity		In vitro	[119]
	DNases	Disrupting mature biofilms		In vitro and animal models	[122,123]
	Dispersin B	Inhibits adherence and attachment		In vitro and animal models	[116,117,124]
	Neutrase from *Bacillus* *Amyloliquefaciens*	Anti-biofilm activity		In vitro	[125]
Bio-surfactants	Mannosylerythritol lipids	Biofilm disruption	Expensive production	In vitro	[126]
Prokaryotes	Cochinmicin from Actinomycetes	Competitive inhibitor of AgrC	Survival of cells during the gastrointestinal transit and their effective delivery to target tissues upon ingestion	In vitro	[95]
	Avellanin from sponges			In vitro	
Chelators	EDTA, EGTA, and TSC	Inhibit biofilm formation	Health hazards, cytotoxic, weakly genotoxic, intracellular metal accumulation, unsuitable for systemic applications	In vitro and limited clinical adjunct use	[127]
Sulfhydryl compounds	DTT, betamercaptoethanol, and cysteine	Inhibit biofilm formation	Skin irritation, organ toxicity, unsuitable for systemic applications	In vitro	[52,128]

Note: ᶲ a virus that specifically targets *S. aureus*. All abbreviations are listed at the end of the manuscript.

**Table 6:** List of the most reported selected medicinal plants and phytochemicals reported to inhibit *S. aureus* biofilm formation.

Plant	Main Bioactive Compound(s)	Molecular Target	Anti-Biofilm Mechanism	Effect on Agr-QS	Experimental Model Used	Effective Concentration	Stage of Development	Reference
*Camellia sinensis*	Catechins, especially EGCG and its derivatives	Bacterial membrane, efflux pumps, amyloid-like biofilm matrix	Membrane disruption, anti-adhesion, anti-biofilm	Interferes with the AgrA response regulator and downregulates RNAIII	*S. aureus* and MRSA clinical isolates	Reported active at concentrations lower than MIC, 10 μg/mL to 60 μg/mL	Preclinical phase	[139,140,141,142]
*Moringa oleifera*	Phytochemicals, fatty acids, including palmitoleic, linolenic, and oleic acids	Quorum sensing and virulence	Inhibits biofilm formation, reduces CFU in biofilms, lowers MIC	Interferes with *agr* locus targeting AgrA or AgrC	*S. aureus* and MRSA from PVC-surface biofilm model	0.5 to 2.0 mg/mL, resulting in up to 99% inhibition	In vitro and early animal stage (feed only)	[140,143,144,145]
*Rosmarinus officinalis*	Diterpene carnosic acid, camphor, micromeric acid, oleanolic acid, ursolic acid	Early attachment and biofilm formation	Inhibits initial attachment, formation, and promotes dispersal of preformed biofilms	Reduces AgrA and RNAIII expression	*S. aureus* and MRSA isolates	0.05 mg/mL (0.1%) extract reported to inhibit biofilm development by 94%	Early preclinical translational stage	[146,147,148]
*Psidium guajava*	Benzyl isocyanate, phenolics, L-5-propylthiomethylhydantoin	Biofilm formation and Quorum sensing	Inhibits biofilm formation	Downregulates *agr*, *icaAD*, and *sarA*	MRSA, other *S. aureus* isolates, and BGM cell-line	100 μg/mL to 1000 μg/mL (sub-minimal inhibitory concentration)	Preclinical phase	[149,150,151,152]
*Eucalyptus globulus*	Essential oil, 1,8-cineole	Early adhesion/attachment	Inhibits initial attachment and adhesion, decreases virulence and biofilm formation	Targets AgrA-AIP (auto-inducing peptide)	MRSA and *S. aureus* isolates	2.5 mg/mL or less	Preclinical phase	[153,154,155]
*Eucalyptus sideroxylon*	Flower extract enriched in phloroglucinols	Biofilm formation and Quorum sensing	Inhibits biofilm formation	Targets Agr system	MRSA and *S. aureus* isolates	0.05 mg/mL achieves up to 95.9% inhibition at sublethal doses	In vitro	[156,157]
*Azadirachta indica*	Crude and methanolic leaf extracts	Disrupting quorum sensing and downregulating virulence	Reduces biofilm formation	Repressing the Agr system	MRSA/MSSA and *S. aureus* isolates	62.5 µg/mL to 125 µg/mL sub-minimum inhibitory concentrations	Preclinical phase	[158,159]
*Curcuma longa*	Curcumin; curcuminoids; diacetyl curcumin	Disrupting quorum sensing and inhibiting swimming and swarming ability	Suppresses biofilm formation, antibiotic synergy, anti-adhesive effects, and photodynamic killing with blue LED	Targets *agrA* and *agrC* operons and RNAIII	MRSA and *S. aureus* isolates	100% inhibition at 20 μM Curcumin plus blue light, 62.5 μg/mL to 125 μg/mL sub-inhibitory concentrations	Preclinical phase	[160,161,162]
*Sanguisorba officinalis*	Triterpenoid saponins; tannins; polyphenolic root extract	Targets the *ica* locus and *agr* system genes	Reduces biofilm formation and density	Interacts with *ica* and *agr* system genes	MRSA and *S. aureus* isolates	256 μg/mL half-maximal inhibitory concentration (IC_50_)	Preclinical phase	[163,164]

Note: preclinical means in vitro and in vivo studies before human testing.

**Table 7:** List of plants with their respective phytochemicals targeting the Agr-QS system with potential anti-biofilm properties.

Scientific Name	Common Name	Part of a Plant	Bioactive Component	Phytochemical	Function	Mechanism	Reference
*Bacopa* *monnieri* (*Plantaginaceae*)	Brahmi (herb)	-	Bacoside A and saponins	Terpenoids	Inhibits microbial adhesion, biofilm formation, and ability to disrupt biofilms	Binding with IcaA	[214,215]
*Cinnamomum* spp. (*Lauraceae*)	Cinnamon	Essential oil and bark	-	-	Cellular shrinkages, cell wall damages, and decreased biofilm densities	-	[216,217]
*Lavandula* *angustifolia* (*Lamiaceae*)	Lavender	Essential oil	Camphor, caryophyllene, eucalyptol, lavendulyl acetate, limonene, linalool, linalyl acetate, cis-ocimene, α-pinene, transocimene, terpinen-4-ol	Terpenoids	Inhibits proliferation and biofilm formation	-	[218,219,220]
*Origanum onites* *and Origanum vulgare* (*Lamiaceae*)	Oregano	Seeds and essential oil	Carvacrol, γ-terpinene, p-cymene, and thymol	Terpenoids	Inhibits biofilm formation and disrupts pre-formed biofilms	-	[219,221]
*Leopoldia comosa* (*Asparagaceae*)	Tassel hyacinth	Bulb	-	-	Inhibits biofilm formation	-	[222]
*Mentha* × *piperita* (*Lamiaceae*)	Pepper mint	Leaves	HCAs, rosmarinic, 1,8-cineole, and menthol	Polyphenol and terpenoids	Inhibits biofilm formation	-	[219,220]
*Ballota nigra* (*Lamiaceae*)	Black horehound	Aerial parts	Phenylpropanoid glycosides and phenylpropanoid derivatives	Flavonoids, glycosides, and terpenes	Inhibits biofilm growth and adherence	Inhibits δ-hemolysin, a small peptide encoded by RNAIII transcript	[1,222,223]
*Sanguisorba* *officinalis* L. (*Rosaceae*)	Great burnet	Dried roots	Saponins	Terpenoids and tannins	Anti-biofilm activity	*ica*-dependent manner	[163,202,203]
*Juglans regia* L. (*Juglandaceae*)	Walnut	Immature fruits and leaves	Naphtoquinones	Quinones, polyphenols and flavonoids	Anti-biofilm activity	-	[222]
*Rhodomyrtus* *tomentosa* (*Myrtaceae*)	Kemunting, rose, myrtle	Leaf	Rhodomyrtone	Flavonoid	Inhibits adherence and biofilm formations	Possible cure for mastitis, even better than vancomycin	[224]
*Rosa damascene* (*Rosaceae*)	Damask rose	Flower	-	-	Eradicates biofilms	-	[225]
*Rosa canina* (*Rosaceae*)	Rosehip	Fruit	-	Flavonoids and polyphenols	Inhibits biofilm formation	Inhibition of exopolysaccharides	[222,226]
*Sambucus nigra* and *Sambucus* *ebulus* (*Adoxaceae*)	Elder and dwarf elder	Leaves and stems	-	Flavonoids	Inhibits biofilm formation	Inhibits δ-hemolysin, a small peptide encoded by RNAIII transcript	[1,227]
*Cyclamen* *hederifolium* (*Myrsinaceae*)	Ivy-leaved cyclamen	Tubers	Saponins	Terpenoids	Inhibits biofilm formation	Inhibits δ-hemolysin, a small peptide encoded by RNAIII transcript	[222,227]
*Ocimum sanctum* (*Lamiaceae*)	Basil	Leaves	-	Eugenol and tannins	Inhibits biofilm formation	-	[193,228,229]
*Lonicera alpigena* (*Caprifoliaceae*)	Alpine honeysuckle	Woody parts and leaves	-	-	Inhibits biofilm formation	Inhibits δ-hemolysin, a small peptide encoded by RNAIII transcript	[222,227]
*Nigella sativa* (*Ranunculaceae*)	Black cumin	Seed oil	Thymoquinone	Quinones	Inhibits biofilm formation	-	[230,231]
*Castanea sativa* (*Fagaceae*)	European chestnut	Leaves	Ursene and oleanene	Flavonoids and terpenoids	Inhibits biofilm formation	Inhibits the *agr* system	[222,227]
*Malva sylvestris* (*Malvaceae*)	Common mallow	Stems and flowers	Menthol and sorbitol	Terpene	Anti-biofilm activity	Used in commercial and mouth rinses	[222,227]
*Thymus vulgaris* (*Lamiaceae*)	Red thyme	Essential oils	Thymol	Terpenoids	Anti-biofilm activity	-	[232,233,234]
*Alcea rosea L.* (*Malvaceae*)	Hollyhock	Leaves, stems, flowers, and roots	Menthol	Terpene	Anti-biofilm activity	Inhibits δ-hemolysin, a small peptide encoded by the RNAIII transcript	[1,227]
*Hydrastis* *canadensis* (*Ranunculaceae*)	Goldenseal	Leaves	Berberine and Mycopyranone: A 8,8′-binaphthopyranone	Alkaloids	Anti-biofilm activity	Inhibits the agr QS system by blocking signal transduction of the AgrCA two-component system	[235,236]
*Solanum nigrum* (*Solanacae*)	Black nightshade	Leaves	-	-	Anti-biofilm activity	-	[228,230]
*Rhanterium* *suaveolens* (*Asteraceae*)	Arfej (shrub)	Essential oil	Carvacrol, linalool, and citrals	Polyphenols	Anti-biofilm activity	-	[237]
*Rosmarinus* *officinalis* (*Lamiaceae*)	Rosemary	Leaves	Pinene, camphor, micromeric acid, oleanolic acid, and ursolic acid	Terpenoids	Inhibits biofilm formation and disrupts pre-formed biofilms	-	[174,175,176,238,239,240]
*Cananga odorata* (*Annonaceae*)	Fragrant cananga	Essential oils	p-cresyl methyl ether, linalool, geranyl acetate, geraniol, eucalyptol	Polyphenols and terpenoids	Anti-biofilm activity	-	[218,232]
*Rubus ulmifolius* (*Rosaceae*)	Elm-leaf blackberry	Leaves, stem, and roots	Ellagic acid derivatives	Polyphenols and glycosides	Anti-biofilm activity	Inhibits δ-hemolysin, a small peptide encoded by RNAIII transcript	[222,227,241,242,243]
*Melissa officinalis* (*Lamiaceae*)	Lemon balm	Essential oil	Citrals (geranial + neral, citronellal, limonene, geraniol, β-caryophyllene, β-caryophyllene oxide, and germacrene D)	Terpenoids	Anti-biofilm activity	-	[218,219]
*Cocculus trilobus* (*Menispermaceae*)	Queen coralbead	Rhizome	Isoquinoline	Alkaloids and quinones	Inhibits microbial adhesion and biofilm formation	-	[244]
*Coriandrum* *sativum* L. (*Apiaceae*)	Coriander	Seeds and essential oil	p-cymene, g-terpinene, linalool, geranyl acetate	Terpenoids	Anti-biofilm activity	-	[219,220,245,246]
*Zanthoxylum* *armatum* (*Rutaceae*)	Winged prickly ash	Fruit	-	Alkaloids and others	Anti-biofilm activity	-	[247]
*Ficus sansibarica* (*Moraceae*)	Knobbly fig	Fruits, leaves, and stem bark	5,7,4′-trihydroxyflavan-3-ol and isovitexin	Flavonoids and triterpenes	Inhibits microbial adhesion and biofilm formation	-	[248,249]
*Marrubium* *vulgare* (*Lamiaceae*)	White horehound	Roots, leaves, stem, and flowers	-	Terpenes, sterols, and flavonoids	Inhibits microbial adhesion and biofilm formation	Inhibits δ-hemolysin, a small peptide encoded by the RNAIII transcript	[222,227]
*Jatropha curcas* (*Euphorbiaceae*)	Purging nut	Pressed cake of whole plant and seed oil	Saponins, linoleic acid, and oleic acid	Alkaloids and polyphenols	Biofilm inhibition and degradation	-	[208,250]
*Pimpinella* *anisum* L. (*Apiaceae*)	Anise	Seeds and essential oil	(*E*)-anethole and estragol	Phenyl propanoids	Anti-biofilm activity	-	[219,220]
*Dischidia* *rafflesiana* (*Apocynaceae*)	Ant plant	Polyherbal formulations	-	-	Anti-biofilm activity	-	[246,251]
*Krameria argentea* (*Krameriaceae*)	Brazilian Rhatany	Roots	Chelerythrine, sanguinarine, dihydroxybenzofuran, and proanthocyanidin	Alkaloids and quinones	Anti-biofilm activity	Interferes with the *arg*-QS system	[243]
*Lawsonia inermis* (*Lythraceae*)	Henna tree	Leaves	Lawsone	Quinones	Anti-biofilm activity	-	[246,252]
*Olea europaea* L. (*Oleaceae*)	Olives	Leaves	Oleuropein	Polyphenols and flavonoids	Anti-biofilm activity	-	[253]
*Glycyrrhiza* *glabra* (*Fabaceae*)	Liquorice	Root	Glycyrrhizin, triterpinoid saponin, and glabridin	Terpenoids	Preventing biofilm formation and adherence	Interferes with the arg-QS system and inhibits exotoxin production	[246,254]
*Leopoldia comosa* (*Hyacinthaceae*)	Tassel grape hyacinth	Bulb	-	-	Preventing biofilm formation and adherence	-	[222]
*Annona* *senegalensis* (*Annonaceae*)	Wild custard apple	Seeds	N-cerotoyltryptamine, asimicin, and ent-19-carbomethoxykauran-17-oic acid	Polyphenols	Anti-biofilm activity	Interferes with the arg-QS system	[207]
*Quercus cerris* L., (*Fagaceae*)	Oak	Leaves, stem, and fruit	-	-	Anti-biofilm activity	Inhibits δ-hemolysin, a small peptide encoded by the RNAIII transcript	[222,227,255]
*Orostachys japonicus* (*Crassulaceae*)	Rock pine	Whole plant	-	Quinones	Inhibits cell-surface attachment	Downregulation of the *psm-mec* gene	[209]
*Phyllanthus* *emblica* (*Phyllanthaceae*)	Indian Gooseberry	Fruits	Gallic acid	Polyphenols	Inhibits cell-surface attachment	-	[246,256]
*Melaleuca* *alternifolia* (*Myrtaceae*)	Tea tree	Essential oil	4-Terpineol and terpinolene	Terpenoids	Inhibits biofilm adhesion	Alters threonine, purine, pyrimidine, and amino acid biosynthesis pathways	[257,258]
*Terminalia* *bellirica* (*Combretaceae*)	Beleric nut tree (Baheda)	Fruit	Termilignan, thannilignan, and anolignan	Tannins	Inhibits biofilm formation	-	[246,259]
*Vanilla planifolia* (*Orchidaceae*)	Vanilla	Pods and essential oil	Ethylvanillin, 4-hydroxybenzaldehyde, methyl anisate, 4-hydroxybenzyl methyl ether, piperonal, vanillic acid, vanillin, carvacrol, and thymol	Terpenoids, flavonoids, and polyphenols	Inhibits biofilm formation	Interferes with the agr-QS system	[202,218]
*Dendrobium chrysotoxum* (*Orchidaceae*)	Fried-egg orchid	Whole plant	Erianin isovitexin and parthenolide	Flavonoids	Inhibits biofilm adhesion	Interferes with the *agr*-QS system	[260]
*Allium sativum* (*Amaryllidaceae*)	Garlic	Essential oil	Allicin	Alkaloids	Inhibits biofilm formation	Downregulates the expression of *icaA* and interferes with *agr* expression	[261,262]
*Vaccinium macrocarpon* (*Ericaceae*)	American cranberry	Leaves and fruit	Urell R and proanthocyanins	Quinones and polyphenols	Inhibits microbial adhesion and biofilm formation	Non-toxic, cyto- compatible metabolites	[243,263]
*Myristica fragrans* (*Myristicaceae*)	Nutmeg	Seed essential oil	Eugenol, isoelemicin, isoeugenol, methoxy eugenol, myristic acid, myristicin, kayeassamin A, surangin C, theraphin B,	Polyphenols and alkaloids	Inhibits biofilm formation	-	[246]
*Arundo donax* (*Poaceae*)	Giant reed	Reed nodes	Bufotenidine and gramine	Alkaloids	Inhibits biofilm formation and disrupts already established biofilms	-	[222]
*Citrus* × *paradisi* (*Rutaceae*)	Grapefruit	Seeds and essential oil	Naringenin	Flavonoid	Inhibits microbial adhesion and biofilm formation	Reduces *agrA* and *hla*	[264,265]
*Spondias* *purpurea* (*Anacardiaceae*)	Spanish Plum	Leaves and fruit juice pulp	-	Terpenoids and flavonoids, polyphenols	Inhibits biofilm formation	Interferes with the *agr*-QS system	[211,266]
*Aesculus* *hippocastanum* (*Sapindaceae*)	Horse chestnut	Whole plant	Chelerythrine, sanguinarine, umbelliferone-3, aesculetin, dihydroxybenzofuran, and proanthocyanidin	Quinones and polyphenols	Inhibits microbial adhesion and biofilm formation	-	[241,243]
*Moringa* *stenopetala* (*Moringaceae*)	Cabbage tree/ African horse radish tree	Leaves and seeds	-	-	Inhibits biofilm formation	-	[205,210]
*Vetiveria zizanioides* (*Poaceae*)	Vetiver (Khus)	Roots	β-vetivenene, vetiselinenol, isovalencenol, vetivenic acid, α-vetivone, and β-vetivone	Terpenoids and polyphenols	Inhibits microbial adhesion and biofilm formation	Inhibits EPS and α-hemolysin toxin production via the *agr*-QS system	[213,267,268]
*Citrus sinensis* (*Rutaceae*)	Sweet orange	Fruits and essential oils from the peel	Limonene, myrcene, α-farnesene, γ-terpinene, α-pinene, and sabinene	Terpenes	Inhibits biofilm formation	-	[213,269]
*Pogostemon cablin* (*Lamiaceae*)	Patchouli	Whole plant	α-guaiene, β-caryophyllene, δ-cadinene, pogostol, patchoulol, seychellene, α- and β-patchoulene	Terpenoids, flavonoids, glycosides	Biofilm eradication	Upregulates biofilm-related bacterial genes *luxR* (inhibitor for the *arg*-QS system)	[246,270]
*Hymenocallis littoralis* (*Amaryllidaceae*)	Spider lily	Leaves	4-methylesculetin, methylisoeugenol, Quercetin 5,7,3′,4′-tetramethyl ether 3-rutinoside	Polyphenols and flavonoids	Inhibits microbial adhesion and biofilm formation	Blocks the active site residues of adhesion proteins	[271,272]
*Cymbopogon flexuosus* (*Poaceae*)	Lemon grass	Essential oil	Citral and β-Geranial	Aliphatic aldehydes	Inhibits microbial adhesion and proliferation to disrupt biofilm matrix	Inhibits PIA and *arg*-QS system	[273,274]
*Dracaena cochinchinensis* (*Asparagaceae*)	Chinese dragon’s blood (red resin)	Resin powder	Homo isoflavans and homo isoflavanones	Flavonoids	Disrupts biofilm	Downregulates biofilm regulatory genes *saeR*, *saeS*, and *hla*	[275,276]
*Duabanga* *grandiflora* (*Lythraceae*)	Duabanga	Leaves	F-10 fraction	Alkaloids, tannins, saponins, steroids, glycosides, and flavonoids	Inhibits cell-surface attachment and biofilm formation	Interfere with the agr-QS system and competitive inhibitor of PBP2a	[277,278]
*Cymbopogon* *nardus* (*Poaceae*)	Citronella grass	Essential oil	Geraniol and citronellal	Terpenes	Inhibits microbial adhesion and proliferation	Non-toxic, cytocompatible metabolites as an alternative for future mouthwashes formulations	[279,280]
*Chelidonium* *majus* (*Papaveraceae*)	Great celandine	Whole plant	Chelerythrine, sanguinarine, dihydroxybenzofuran, and proanthocyanidin	Alkaloids	Inhibits cell-surface attachment and biofilm formation	-	[236,243,281]
*Ocimum* *gratissimum* (*Lamiaceae*)	Clove basil	Essential oil and leaves	Eugenol, 1,8-cineole, α-terpineol, γ-terpinene	Alkaloids and terpenes	Inhibits cell-surface attachment and biofilm formation		[282,283]

Note: All abbreviations are listed at the end of the manuscript.

## Data Availability

No new data were created or analyzed in this study. Data sharing is not applicable to this article.

## Abbreviations

The following abbreviations were used in the manuscript.

MRSA	Methicillin-resistant *S. aureus*
MSSA	Methicillin-susceptible *S. aureus*
CPK	Creatine phosphokinase
PVL	Panton–Valentine leucocidin
EVD	External ventricular drains
MSCRAMMs	Microbial surface components recognizing adhesive matrix molecules
TSST-1	Toxic shock syndrome toxin 1
ETs	Exfoliative toxins
MTSS	Menstrual toxic shock syndrome
NMTSS	Non-menstrual TSS
SSSS	Staphylococcal scalded skin syndrome
SFD	Staphylococcal food-borne diseases
EPSs	Exopolysaccharides
PIA	Polysaccharide intercellular adhesion
QS	Quorum sensing
AIPs	Auto-inducing peptides
PSMs	Phenol soluble modulins
ORFs	Open reading frames
LA-MRSA	Livestock-associated methicillin-resistant *S. aureus*
CA-MRSA	Community-associated methicillin-resistant *S. aureus*
VRSA	Vancomycin-resistant *S. aureus*
CAGR	Compound annual growth rate
EDTA	Ethylenediamine tetraacetic acid
EGTA	Ethylene glycol tetra acetic acid
TSC	Tri-sodium citrate
DTT	Dithiothreitol
EC	Epicatechin
EGC	Epigallocatechin
ECG	Epicatechin gallate
EGCG	Epigallocatechin gallate
PVC	Polyvinyl Chloride plastic
GC-MS	Gas chromatography–mass spectrometry
MIC	Minimum inhibitory concentration
NMR	Nuclear magnetic resonance
UPLC	Ultra-performance liquid chromatography
BIC	Benzyl isocyanate
LC-FTMS	Liquid chromatography Fourier transform mass spectrometry
SEM	Scanning electron microscopy

## References

[1] Li J., Liu D., Tian X., Koseki S., Chen S., Ye X., Ding T. (2019). Novel antibacterial modalities against methicillin resistant *Staphylococcus aureus* derived from plants. Crit. Rev. Food Sci. Nutr..

[2] Sollid J.U.E., Furberg A., Hanssen A., Johannessen M. (2014). *Staphylococcus aureus*: Determinants of human carriage. Infect. Genet. Evol..

[3] Hindieh P., Yaghi J., Assaf J.C., Chokr A., Atoui A., Tzenios N., Louka N., Khoury A.E. (2025). Emerging Multimodal Strategies for Bacterial Biofilm Eradication: A Comprehensive Review. Microorganisms.

[4] Kiedrowski M.R., Horswill A.R. (2011). New approaches for treating staphylococcal biofilm infections. Ann. N. Y. Acad. Sci..

[5] Høiby N., Ciofu O., Johansen H.K., Song Z.j., Moser C., Jensen P.Ø., Molin S., Givskov M., Tolker-Nielsen T., Bjarnsholt T. (2011). The clinical impact of bacterial biofilms. Int. J. Oral Sci..

[6] Jamal M., Ahmad W., Andleeb S., Jalil F., Imran M., Nawaz M.A., Hussain T., Ali M., Rafiq M., Kamil M.A. (2018). Bacterial biofilm and associated infections. J. Chin. Med. Assoc..

[7] Tuchscherr L., Löffler B., Buzzola F.R., Sordelli D.O. (2010). *Staphylococcus aureus* adaptation to the host and persistence: Role of loss of capsular polysaccharide expression. Future Med..

[8] Zaatout N., Ayachi A., Kecha M. (2019). Interaction of primary mammary bovine epithelial cells with biofilm-forming staphylococci associated with subclinical bovine mastitis. Iran. J. Vet. Res..

[9] Pietrocola G., Nobile G., Rindi S., Speziale P. (2017). *Staphylococcus aureus* manipulates innate immunity through own and host-expressed proteases. Front. Cell. Infect. Microbiol..

[10] Parvizi J., Pawasarat I.M., Azzam K.A., Joshi A., Hansen E.N., Bozic K.J. (2010). Periprosthetic joint infection: The economic impact of methicillin-resistant infections. J. Arthroplast..

[11] Song X., Perencevich E., Campos J., Short B.L., Singh N. (2010). Clinical and economic impact of methicillin-resistant *Staphylococcus aureus* colonization or infection on neonates in intensive care units. Infect. Control Hosp. Epidemiol..

[12] Pesavento G., Maggini V., Maida I., Nostro A.L., Calonico C., Sassoli C., Perrin E., Fondi M., Mengoni A., Chiellini C. (2016). Essential oil from *Origanum vulgare* completely inhibits the growth of multidrug-resistant cystic fibrosis pathogens. Nat. Prod. Commun..

[13] Solórzano-Santos F., Miranda-Novales M.G. (2012). Essential oils from aromatic herbs as antimicrobial agents. Curr. Opin. Biotechnol..

[14] Tuffs S.W., Haeryfar S.M.M., McCormick J.K. (2018). Manipulation of innate and adaptive immunity by *Staphylococcal* superantigens. Pathogens.

[15] Kadariya J., Smith T.C., Thapaliya D. (2014). *Staphylococcus aureus* and staphylococcal food-borne disease: An ongoing challenge in public health. BioMed Res. Int..

[16] Hennekinne J.-A., De Buyser M.-L., Dragacci S. (2012). *Staphylococcus aureus* and its food poisoning toxins: Characterization and outbreak investigation. FEMS Microbiol. Rev..

[17] Rudd K.E., Johnson S.C., Agesa K.M., Shackelford K.A., Tsoi D., Kievlan D.R., Colombara D.V., Ikuta K.S., Kissoon N., Finfer S. (2020). Global, regional, and national sepsis incidence and mortality, 1990–2017: Analysis for the Global Burden of Disease Study. Lancet.

[18] WHO Sepsis Fact Sheet 2024. https://www.who.int/news-room/fact-sheets/detail/sepsis.

[19] Tobin E.H., Jogu P., Koirala J. Methicillin-Resistant *Staphylococcus aureus*. https://www.ncbi.nlm.nih.gov/books/NBK482221/.

[20] Shoaib M., Aqib A.I., Muzammil I., Majeed N., Bhutta Z.A., Kulyar M.F.-e.-A., Fatima M., Zaheer C.-N.F., Muneer A., Murtaza M. (2023). MRSA compendium of epidemiology, transmission, pathophysiology, treatment, and prevention within one health framework. Front. Microbiol..

[21] Liu C., Bayer A., Cosgrove S.E., Daum R.S., Fridkin S.K., Gorwitz R.J., Kaplan S.L., Karchmer A.W., Levine D.P., Murray B.E. (2011). Clinical Practice Guidelines by the Infectious Diseases Society of America for the Treatment of Methicillin-Resistant *Staphylococcus aureus* Infections in Adults and Children. Clin. Infect. Dis..

[22] Britt N.S., Tirmizi S., Ritchie D.J., Topal J.E., McManus D., Nizet V., Casabar E., Sakoulas G. (2018). Telavancin for refractory MRSA bacteraemia in intermittent haemodialysis recipients. J. Antimicrob. Chemother..

[23] Vanderhaeghen W., Cerpentier T., Adriaensen C., Vicca J., Hermans K., Butaye P. (2010). Methicillin-resistant *Staphylococcus aureus* (MRSA) ST398 associated with clinical and subclinical mastitis in Belgian cows. Vet. Microbiol..

[24] Ha K.N.Y., Vu T.T.T., Nguyen T.T.T., Nguyen H.Q.N., Dang T.N.D. (2025). A retrospective study of the risk factors for linezolid-induced thrombocytopenia in hospitalized patients. MedPharmRes.

[25] Muñoz P., De la Villa S., Martínez-Sellés M., Goenaga M.A., Reviejo-Jaka K., Revillas F.A.L., García-Cuello L., Hidalgo-Tenorio C., Rodríguez-Esteban M.A., Antorrena I. (2021). Linezolid for infective endocarditis: A structured approach based on a national database experience. Medicine.

[26] Shariati A., Dadashi M., Chegini Z., van Belkum A., Mirzaii M., Khoramrooz S.S., Darban-Sarokhalil D. (2020). The global prevalence of Daptomycin, Tigecycline, Quinupristin/Dalfopristin, and Linezolid-resistant *Staphylococcus aureus* and coagulase-negative staphylococci strains: A systematic review and meta-analysis. Antimicrob. Resist. Infect. Control.

[27] Kobayashi T., Ando T., Streit J., Sekar P. (2019). Current Evidence on Oral Antibiotics for Infective Endocarditis: A Narrative Review. Cardiol. Ther..

[28] Skiest D.J. (2006). Treatment failure resulting from resistance of *Staphylococcus aureus* to daptomycin. J. Clin. Microbiol..

[29] Fiore M., Alfieri A., Fiore D., Iuliano P., Spatola F.G., Limone A., Pezone I., Leone S. (2025). Use of Daptomycin to Manage Severe MRSA Infections in Humans. Medicine.

[30] Bhavnani S.M., Rubino C.M., Ambrose P.G., Drusano G.L. (2010). Daptomycin exposure and the probability of elevations in the creatine phosphokinase level: Data from a randomized trial of patients with bacteremia and endocarditis. Clin. Infect. Dis. Off. Publ. Infect. Dis. Soc. Am..

[31] Tally F.P., DeBruin M.F. (2000). Development of daptomycin for gram-positive infections. J. Antimicrob. Chemother..

[32] Marty F.M., Yeh W.W., Wennersten C.B., Venkataraman L., Albano E., Alyea E.P., Gold H.S., Baden L.R., Pillai S.K. (2006). Emergence of a clinical daptomycin-resistant *Staphylococcus aureus* isolate during treatment of methicillin-resistant *Staphylococcus aureus* bacteremia and osteomyelitis. J. Clin. Microbiol..

[33] Zamoner W., Prado I.R.S., Balbi A.L., Ponce D. (2019). Vancomycin dosing, monitoring and toxicity: Critical review of the clinical practice. Clin. Exp. Pharmacol. Physiol..

[34] Niforatos J.D., Hinson J.S., Rothman R.E., Cosgrove S.E., Dzintars K., Klein E.Y. (2025). Methicillin-resistant *Staphylococcus aureus* and Vancomycin Prescribing in the Emergency Department: A Single-center Study Assessing Antibiotic Prescribing. J. Am. Coll. Emerg. Phys. Open.

[35] Keikha M., Karbalaei M. (2024). Global distribution of heterogeneous vancomycin-intermediate *Staphylococcus aureus* strains (1997–2021): A systematic review and meta-analysis. J. Glob. Antimicrob. Resist..

[36] Sievert D.M., Rudrik J.T., Patel J.B., McDonald L.C., Wilkins M.J., Hageman J.C. (2008). Vancomycin-resistant *Staphylococcus aureus* in the United States, 2002–2006. Clin. Infect. Dis. Off. Publ. Infect. Dis. Soc. Am..

[37] Zhang S., Sun X., Chang W., Dai Y., Ma X. (2015). Systematic review and meta-analysis of the epidemiology of vancomycin-intermediate and heterogeneous vancomycin-intermediate *Staphylococcus aureus* isolates. PLoS ONE.

[38] Deotale V., Mendiratta D.K., Raut U., Narang P. (2010). Inducible clindamycin resistance in *Staphylococcus aureus* isolated from clinical samples. Indian J. Med. Microbiol..

[39] Fomda B.A., Peer M.A., Zahoor D., Thokar M.A., Nasir R.A. (2010). Phenotypic detection of constitutive and inducible clindamycin resistance in clinical isolates of *Staphylococcus aureus* and coagulase negative *Staphylococcus* on routine susceptibility plate. J. Commun. Dis..

[40] Addis Z., Aschale Y., Fenta A., Teffera Z.H., Melkamu A., Tigab A., Dilnessa T. (2025). Methicillin and inducible clindamycin resistance in clinical *Staphylococcus aureus* isolates: A cross-sectional study from Northwest Ethiopia. Front. Microbiol..

[41] Werner G., Cuny C., Schmitz F.J., Witte W. (2001). Methicillin-resistant, quinupristin-dalfopristin-resistant *Staphylococcus aureus* with reduced sensitivity to glycopeptides. J. Clin. Microbiol..

[42] Pharm U. (2022). ToExamTable Blog Summary with Recent VISA/VRSA Data and Therapeutic Options: “Vancomycin-Resistant *Staphylococcus aureus*: Emergence and Treatment Options”. https://blog.unmc.edu/infectious-disease/2022/11/03/pharmtoexamtable-vancomycin-resistant-staphylococcus-aureus-emergence-and-treatment/.

[43] Coelho C., de Lencastre H., Aires-de-Sousa M. (2017). Frequent occurrence of trimethoprim-sulfamethoxazole hetero-resistant *Staphylococcus aureus* isolates in different African countries. Eur. J. Clin. Microbiol. Infect. Dis. Off. Publ. Eur. Soc. Clin. Microbiol..

[44] Abd El-Hamid M.I., Bendary M.M., Merwad A.M.A., Elsohaby I., Mohammad Ghaith D., Alshareef W.A. (2019). What is behind phylogenetic analysis of hospital-, community- and livestock-associated methicillin-resistant *Staphylococcus aureus*? *Transbound*. Emerg. Dis..

[45] Van Acker H., Van Dijck P., Coenye T. (2014). Molecular mechanisms of antimicrobial tolerance and resistance in bacterial and fungal biofilms. Trends Microbiol..

[46] McCarthy H., Rudkin J.K., Black N.S., Gallagher L., O’Neill E., O’Gara J.P. (2015). Methicillin resistance and the biofilm phenotype in *Staphylococcus aureus*. Front. Cell. Infect. Microbiol..

[47] Lewis K. (2008). Multidrug tolerance of biofilms and persister cells. Curr. Top. Microbiol. Immunol..

[48] Otto M. (2014). Physical stress and bacterial colonization. FEMS Microbiol. Rev..

[49] Zaatout N., Ayachi A., Kecha M. (2020). *Staphylococcus aureus* persistence properties associated with bovine mastitis and alternative therapeutic modalities. J. Appl. Microbiol..

[50] Archer N.K., Mazaitis M.J., Costerton J.W., Leid J.G., Powers M.E., Shirtliff M.E. (2011). *Staphylococcus aureus* biofilms: Properties, regulation, and roles in human disease. Virulence.

[51] Kraushaar B., Fetsch A. (2014). First description of PVL-positive methicillin-resistant *Staphylococcus aureus* (MRSA) in wild boar meat. Int. J. Food Microbiol..

[52] Arciola C.R., Campoccia D., Ravaioli S., Montanaro L. (2015). Polysaccharide intercellular adhesin in biofilm: Structural and regulatory aspects. Front. Cell. Infect. Microbiol..

[53] Boles B.R., Horswill A.R. (2008). Agr-mediated dispersal of *Staphylococcus aureus* biofilms. PLoS Pathog..

[54] Tan L., Li S.R., Jiang B., Hu X.M., Li S. (2018). Therapeutic targeting of the *Staphylococcus aureus* accessory gene regulator (*agr*) system. Front. Microbiol..

[55] Traber K.E., Lee E., Benson S., Corrigan R., Cantera M., Shopsin B., Novick R.P. (2008). Agr function in clinical *Staphylococcus aureus* isolates. Microbiology.

[56] Le K.Y., Otto M. (2015). Quorum-sensing regulation in staphylococci—An overview. Front. Microbiol..

[57] Reffuveille F., Josse J., Vallé Q., Gangloff C., Gangloff S.C. (2017). *Staphylococcus aureus* biofilms and their impact on the medical field. Rise Virulence Antibiot. Resist. Staphylococcus Aureus.

[58] Josse J., Velard F., Gangloff S.C. (2015). *Staphylococcus aureus* vs. osteoblast: Relationship and consequences in osteomyelitis. Front. Cell. Infect. Microbiol..

[59] Otto M. (2013). Staphylococcal infections: Mechanisms of biofilm maturation and detachment as critical determinants of pathogenicity. Annu. Rev. Med..

[60] Nguyen H.T.T., Nguyen T.H., Otto M. (2020). The staphylococcal exopolysaccharide PIA—Biosynthesis and role in biofilm formation, colonization, and infection. Comput. Struct. Biotechnol. J..

[61] Lister J.L., Horswill A.R. (2014). *Staphylococcus aureus* biofilms: Recent developments in biofilm dispersal. Front. Cell. Infect. Microbiol..

[62] Sugimoto S., Sato F., Miyakawa R., Chiba A., Onodera S., Hori S., Mizunoe Y. (2018). Broad impact of extracellular DNA on biofilm formation by clinically isolated Methicillin-resistant and -sensitive strains of *Staphylococcus aureus*. Sci. Rep..

[63] Rohde H., Burandt E.C., Siemssen N., Frommelt L., Burdelski C., Wurster S., Scherpe S., Davies A.P., Harris L.G., Horstkotte M.A. (2007). Polysaccharide intercellular adhesin or protein factors in biofilm accumulation of *Staphylococcus epidermidis* and *Staphylococcus aureus* isolated from prosthetic hip and knee joint infections. Biomaterials.

[64] O’Neill E., Pozzi C., Houston P., Humphreys H., Robinson D.A., Loughman A., Foster T.J., O’Gara James P. (2008). A Novel *Staphylococcus aureus* Biofilm Phenotype Mediated by the Fibronectin-Binding Proteins, FnBPA and FnBPB. J. Bacteriol..

[65] Geoghegan J.A., Corrigan R.M., Gruszka D.T., Speziale P., O’Gara J.P., Potts J.R., Foster T.J. (2010). Role of Surface Protein SasG in Biofilm Formation by *Staphylococcus aureus*. J. Bacteriol..

[66] Speziale P., Pietrocola G., Foster T.J., Geoghegan J.A. (2014). Protein-based biofilm matrices in Staphylococci. Front. Cell. Infect. Microbiol..

[67] Kavanaugh Jeffrey S., Flack Caralyn E., Lister J., Ricker Erica B., Ibberson Carolyn B., Jenul C., Moormeier Derek E., Delmain Elizabeth A., Bayles Kenneth W., Horswill Alexander R. (2019). Identification of Extracellular DNA-Binding Proteins in the Biofilm Matrix. mBio.

[68] Lasa I., Penadés J.R. (2006). Bap: A family of surface proteins involved in biofilm formation. Res. Microbiol..

[69] Cue D., Lei M.G., Luong T.T., Kuechenmeister L., Dunman P.M., O’Donnell S., Rowe S., O’Gara J.P., Lee C.Y. (2009). Rbf promotes biofilm formation by *Staphylococcus aureus* via repression of *icaR*, a negative regulator of *icaADBC*. J. Bacteriol..

[70] Abisado R.G., Benomar S., Klaus J.R., Dandekar A.A., Chandler J.R. (2018). Bacterial quorum sensing and microbial community interactions. mBio.

[71] Passos da Silva D., Schofield M.C., Parsek M.R., Tseng B.S. (2017). An update on the sociomicrobiology of quorum sensing in gram-negative biofilm development. Pathogens.

[72] Xie Y., He Y., Gehring A., Hu Y., Li Q., Tu S.-I., Shi X. (2011). Genotypes and toxin gene profiles of *Staphylococcus aureus* clinical isolates from China. PLoS ONE.

[73] Bardiau M., Caplin J., Detilleux J., Graber H., Moroni P., Taminiau B., Mainil J.G. (2016). Existence of two groups of *Staphylococcus aureus* strains isolated from bovine mastitis based on biofilm formation, intracellular survival, capsular profile and agr-typing. Vet. Microbiol..

[74] Fechter P., Caldelari I., Lioliou E., Romby P. (2014). Novel aspects of RNA regulation in *Staphylococcus aureus*. FEBS Lett..

[75] Nichol K.A., Adam H.J., Hussain Z., Mulvey M.R., McCracken M., Mataseje L.F., Thompson K., Kost S., Lagacé-Wiens P.R., Hoban D.J. (2011). Comparison of community-associated and health care-associated methicillin-resistant *Staphylococcus aureus* in Canada: Results of the CANWARD 2007-2009 study. Diagn. Microbiol. Infect. Dis..

[76] Mohsenzadeh M., Ghazvini K., Azimian A. (2015). Frequency of specific *agr* groups and antibiotic resistance in *Staphylococcus aureus* isolated from bovine mastitis in the northeast of Iran. Vet. Res. Forum Int. Q. J..

[77] Zhang Y., Xu D., Shi L., Cai R., Li C., Yan H. (2018). Association between *agr* type, virulence factors, biofilm formation and antibiotic resistance of *Staphylococcus aureus* isolates from pork production. Front. Microbiol..

[78] Cafiso V., Bertuccio T., Santagati M., Demelio V., Spina D., Nicoletti G., Stefani S. (2007). Agr-Genotyping and transcriptional analysis of biofilm-producing *Staphylococcus aureus*. FEMS Immunol. Med. Microbiol..

[79] Ikonomidis A., Vasdeki A., Kristo I., Maniatis A.N., Tsakris A., Malizos K.N., Pournaras S. (2009). Association of biofilm formation and methicillin-resistance with accessory gene regulator (*agr*) loci in Greek *Staphylococcus aureus* clones. Microb. Pathog..

[80] Khoramrooz S.S., Mansouri F., Marashifard M., Malek Hosseini S.A., Akbarian Chenarestane-Olia F., Ganavehei B., Gharibpour F., Shahbazi A., Mirzaii M., Darban-Sarokhalil D. (2016). Detection of biofilm related genes, classical enterotoxin genes and *agr* typing among *Staphylococcus aureus* isolated from bovine with subclinical mastitis in southwest of Iran. Microb. Pathog..

[81] Ventola C.L. (2015). The antibiotic resistance crisis: Part 1: Causes and threats. Pharm. Ther..

[82] Seattle. Methicillin-Resistant *Staphylococcus aureus* Market Size Is Estimated to Reach US$ 1,327.9 Million by 2027-Press Release. https://www.pharmiweb.com/press-release/2021-03-25/methicillin-resistant-staphylococcus-aureus-market-size-is-estimated-to-reach-us-1-3279-million-by.

[83] Manoharadas S., Altaf M., Alrefaei A.F., Devasia R.M., Badjah Hadj A.Y.M., Abuhasil M.S.A. (2021). Concerted dispersion of *Staphylococcus aureus* biofilm by bacteriophage and ‘green synthesized’ silver nanoparticles. RSC Adv..

[84] Wypij M., Jędrzejewski T., Trzcińska-Wencel J., Ostrowski M., Rai M., Golińska P. (2021). Green synthesized silver nanoparticles: Antibacterial and anticancer activities, biocompatibility, and analyses of surface-attached proteins. Front. Microbiol..

[85] Zhou Y., Zhao R., Ma B., Gao H., Xue X., Qu D., Li M., Meng J., Luo X., Hou Z. (2016). Oligomerization of RNAIII-Inhibiting Peptide Inhibits Adherence and Biofilm Formation of Methicillin-Resistant *Staphylococcus aureus* In Vitro and In Vivo. Microb. Drug Resist..

[86] Ma B., Zhou Y., Li M., Yu Q., Xue X., Li Z., Da F., Hou Z., Luo X. (2015). RIP-V improves murine survival in a sepsis model by down-regulating RNAIII expression and α-hemolysin release of methicillin-resistant *Staphylococcus aureus*. Die Pharm..

[87] Salam A.M., Quave C.L. (2018). Targeting virulence in *Staphylococcus aureus* by chemical inhibition of the accessory gene regulator system in vivo. mSphere.

[88] Sully E.K., Malachowa N., Elmore B.O., Alexander S.M., Femling J.K., Gray B.M., DeLeo F.R., Otto M., Cheung A.L., Edwards B.S. (2014). Selective chemical inhibition of agr quorum sensing in *Staphylococcus aureus* promotes host defense with minimal impact on resistance. PLoS Pathog..

[89] Pant N., Miranda-Hernandez S., Rush C., Warner J., Eisen D.P. (2022). Effect of savirin in the prevention of biofilm-related *Staphylococcus aureus* prosthetic joint infection. Front. Pharmacol..

[90] Wallis J.K., Krömker V., Paduch J.-H. (2019). Biofilm challenge: Lactic acid bacteria isolated from bovine udders versus Staphylococci. Foods.

[91] Bouchard D.S., Rault L., Berkova N., Le Loir Y., Even S. (2013). Inhibition of *Staphylococcus aureus* invasion into bovine mammary epithelial cells by contact with live *Lactobacillus casei*. Appl. Environ. Microbiol..

[92] Meroni G., Panelli S., Zuccotti G., Bandi C., Drago L., Pistone D. (2021). Probiotics as Therapeutic Tools against Pathogenic Biofilms: Have We Found the Perfect Weapon?. Microbiol. Res..

[93] Asli A., Brouillette E., Ster C., Ghinet M.G., Brzezinski R., Lacasse P., Jacques M., Malouin F. (2017). Antibiofilm and antibacterial effects of specific chitosan molecules on *Staphylococcus aureus* isolates associated with bovine mastitis. PLoS ONE.

[94] Canovas J., Baldry M., Bojer M.S., Andersen P.S., Grzeskowiak P.K., Stegger M., Damborg P., Olsen C.A., Ingmer H. (2016). Cross-talk between *Staphylococcus aureus* and other Staphylococcal species via the agr quorum sensing system. Front. Microbiol..

[95] Wang B., Muir T.W. (2016). Regulation of Virulence in *Staphylococcus aureus*: Molecular Mechanisms and Remaining Puzzles. Cell Chem. Biol..

[96] O’Flaherty S., Ross R.P., Flynn J., Meaney W.J., Fitzgerald G.F., Coffey A. (2005). Isolation and characterization of two anti-staphylococcal bacteriophages specific for pathogenic *Staphylococcus aureus* associated with bovine infections. Lett. Appl. Microbiol..

[97] Iwano H., Inoue Y., Takasago T., Kobayashi H., Furusawa T., Taniguchi K., Fujiki J., Yokota H., Usui M., Tanji Y. (2018). Bacteriophage ΦSA012 has a broad host range against *Staphylococcus aureus* and effective lytic capacity in a mouse mastitis model. Biology.

[98] Lungren M.P., Christensen D., Kankotia R., Falk I., Paxton B.E., Kim C.Y. (2013). Bacteriophage K for reduction of *Staphylococcus aureus* biofilm on central venous catheter material. Bacteriophage.

[99] Herren S.C., Karau M.J., Guarin Perez S.F., Koscianski C.A., Mandrekar J., Patel R., Bedard N.A. (2025). Activity of Phage and Vancomycin in a Murine *Staphylococcus aureus* Periprosthetic Joint Infection Model Managed With Debridement and Implant Retention. J. Orthop. Res..

[100] Alves D.R., Gaudion A., Bean J.E., Perez Esteban P., Arnot T.C., Harper D.R., Kot W., Hansen L.H., Enright M.C., Jenkins A.T.A. (2014). Combined use of bacteriophage K and a novel bacteriophage to reduce *Staphylococcus aureus* biofilm formation. Appl. Environ. Microbiol..

[101] Klein R.C., Fabres-Klein M.H., de Oliveira L.L., Feio R.N., Malouin F., Ribon Ade O. (2015). A C-type lectin from *Bothrops jararacussu* venom disrupts Staphylococcal biofilms. PLoS ONE.

[102] Mihu M.R., Cabral V., Pattabhi R., Tar M.T., Davies K.P., Friedman A.J., Martinez L.R., Nosanchuk J.D. (2016). Sustained nitric oxide-releasing nanoparticles interfere with methicillin-resistant *Staphylococcus aureus* adhesion and biofilm formation in a rat central venous catheter model. Antimicrob. Agents Chemother..

[103] Masurkar S.A., Chaudhari P.R., Shidore V.B., Kamble S.P. (2012). Effect of biologically synthesised silver nanoparticles on *Staphylococcus aureus* biofilm quenching and prevention of biofilm formation. IET Nanobiotechnol..

[104] Mu H., Tang J., Liu Q., Sun C., Wang T., Duan J. (2016). Potent Antibacterial Nanoparticles against Biofilm and Intracellular Bacteria. Sci. Rep..

[105] Harris M.A., Beenken K.E., Smeltzer M.S., Haggard W.O., Jennings J.A. (2017). Phosphatidylcholine coatings deliver local antimicrobials and reduce infection in a murine model: A preliminary study. Clin. Orthop. Relat. Res.^®^.

[106] Lellouche J., Friedman A., Gedanken A., Banin E. (2012). Antibacterial and antibiofilm properties of yttrium fluoride nanoparticles. Int. J. Nanomed..

[107] Lellouche J., Friedman A., Lahmi R., Gedanken A., Banin E. (2012). Antibiofilm surface functionalization of catheters by magnesium fluoride nanoparticles. Int. J. Nanomed..

[108] Medina-Estrada I., López-Meza J.E., Ochoa-Zarzosa A. (2016). Anti-inflammatory and antimicrobial effects of estradiol in bovine mammary epithelial cells during *Staphylococcus aureus* internalization. Mediat. Inflamm..

[109] Wu X., Wang X., Kang J., Dong A., Yang Y.-W. (2025). Autoinducing Peptide-Mediated Bacterial Keratitis Therapy by Camouflaged *Staphylococcus aureus*. ACS Nano.

[110] Wang J., Nong X.H., Zhang X.Y., Xu X.Y., Amin M., Qi S.H. (2017). Screening of anti-biofilm compounds from marine-derived fungi and the effects of Secalonic acid D on *Staphylococcus aureus* biofilm. J. Microbiol. Biotechnol..

[111] Craigen B., Dashiff A., Kadouri D.E. (2011). The use of commercially available alpha-amylase compounds to inhibit and remove *Staphylococcus aureus* biofilms. Open Microbiol. J..

[112] Kokai-Kun J.F., Chanturiya T., Mond J.J. (2009). Lysostaphin eradicates established *Staphylococcus aureus* biofilms in jugular vein catheterized mice. J. Antimicrob. Chemother..

[113] Index N. Lysostaphin Applications for *Staphylococcus aureus* Infections. https://www.nature.com/nature-index/topics/l4/lysostaphin-applications-for-staphylococcus-aureus-infections.

[114] Zeng P., Zhang P., Chan H.W., Chow S.F., Lam J.K.W., Ip M., Leung S.S.Y. (2024). Storage stability of lysostaphin solution and its pulmonary delivery. Drug Deliv. Transl. Res..

[115] Ibberson C.B., Parlet C.P., Kwiecinski J., Crosby H.A., Meyerholz D.K., Horswill A.R. (2016). Hyaluronan modulation impacts *Staphylococcus aureus* biofilm infection. Infect. Immun..

[116] Fenton M., Keary R., McAuliffe O., Ross R.P., O’Mahony J., Coffey A. (2013). Bacteriophage-Derived Peptidase CHAP_K_ Eliminates and Prevents Staphylococcal Biofilms. Int. J. Microbiol..

[117] Gutiérrez D., Ruas-Madiedo P., Martínez B., Rodríguez A., García P. (2014). Effective Removal of Staphylococcal Biofilms by the Endolysin LysH5. PLoS ONE.

[118] Nasiri Shoeibi Z., Rafiee F., Akrami S., Ghandehari Z., Feizabadi M.M. (2026). Endolysins, Depolymerases, Holins, and VALs: Phage-Derived Proteins for Combating Antimicrobial Resistance. Microb. Drug Resist..

[119] Suresh M.K., Biswas R., Biswas L. (2019). An update on recent developments in the prevention and treatment of *Staphylococcus aureus* biofilms. Int. J. Med. Microbiol..

[120] Mootz J.M., Malone C.L., Shaw L.N., Horswill A.R. (2013). Staphopains modulate *Staphylococcus aureus* biofilm integrity. Infect. Immun..

[121] Loughran A.J., Atwood D.N., Anthony A.C., Harik N.S., Spencer H.J., Beenken K.E., Smeltzer M.S. (2014). Impact of individual extracellular proteases on *Staphylococcus aureus* biofilm formation in diverse clinical isolates and their isogenic *sarA* mutants. Microbiologyopen.

[122] Kaplan J.B., LoVetri K., Cardona S.T., Madhyastha S., Sadovskaya I., Jabbouri S., Izano E.A. (2012). Recombinant human DNase I decreases biofilm and increases antimicrobial susceptibility in *staphylococci*. J. Antibiot..

[123] Deng W., Lei Y., Tang X., Li D., Liang J., Luo J., Liu L., Zhang W., Ye L., Kong J. (2022). DNase inhibits early biofilm formation in *Pseudomonas aeruginosa*- or *Staphylococcus aureus*-induced empyema models. Front. Cell. Infect. Microbiol..

[124] Kaplan J.B., Sukhishvili S.A., Sailer M., Kridin K., Ramasubbu N. (2024). *Aggregatibacter actinomycetemcomitans* Dispersin B: The Quintessential Antibiofilm Enzyme. Pathogens.

[125] Elchinger P.H., Delattre C., Faure S., Roy O., Badel S., Bernardi T., Taillefumier C., Michaud P. (2014). Effect of proteases against biofilms of *Staphylococcus aureus* and *Staphylococcus epidermidis*. Lett. Appl. Microbiol..

[126] Shu Q., Wei T., Lu H., Niu Y., Chen Q. (2020). Mannosylerythritol lipids: Dual inhibitory modes against *Staphylococcus aureus* through membrane-mediated apoptosis and biofilm disruption. Appl. Microbiol. Biotechnol..

[127] Abraham N.M., Lamlertthon S., Fowler V.G., Jefferson K.K. (2012). Chelating agents exert distinct effects on biofilm formation in *Staphylococcus aureus* depending on strain background: Role for clumping factor B. J. Med. Microbiol..

[128] Wu X., Wang Y., Tao L. (2011). Sulfhydryl compounds reduce *Staphylococcus aureus* biofilm formation by inhibiting PIA biosynthesis. FEMS Microbiol. Lett..

[129] Mondal A., Gandhi A., Fimognari C., Atanasov A.G., Bishayee A. (2019). Alkaloids for cancer prevention and therapy: Current progress and future perspectives. Eur. J. Pharmacol..

[130] Gomes F., Martins N., Ferreira I.C.F.R., Henriques M. (2019). Anti-biofilm activity of hydromethanolic plant extracts against *Staphylococcus aureus* isolates from bovine mastitis. Heliyon.

[131] Zacchino S.A., Butassi E., Liberto M.D., Raimondi M., Postigo A., Sortino M. (2017). Plant phenolics and terpenoids as adjuvants of antibacterial and antifungal drugs. Phytomedicine.

[132] Swolana D., Kępa M., Kabała-Dzik A., Dzik R., Wojtyczka R.D. (2021). Sensitivity of Staphylococcal biofilm to selected compounds of plant origin. Antibiotics.

[133] Dong G., Liu H., Yu X., Zhang X., Lu H., Zhou T., Cao J. (2018). Antimicrobial and anti-biofilm activity of tannic acid against *Staphylococcus aureus*. Nat. Prod. Res..

[134] Maisetta G., Batoni G., Caboni P., Esin S., Rinaldi A.C., Zucca P. (2019). Tannin profile, antioxidant properties, and antimicrobial activity of extracts from two Mediterranean species of parasitic plant *Cytinus*. BMC Complement. Altern. Med..

[135] Schestakow A., Guth M.S., Eisenmenger T.A., Hannig M. (2021). Evaluation of anti-biofilm activity of mouthrinses containing tannic acid or chitosan on dentin in situ. Molecules.

[136] Ze-Feng W., Jia L., Yong-An Y., Hai-Liang Z. (2020). A Review: The anti-inflammatory, anticancer and antibacterial properties of four kinds of licorice flavonoids isolated from licorice. Curr. Med. Chem..

[137] Feng D., Zhang A., Yang Y., Yang P. (2020). Coumarin-containing hybrids and their antibacterial activities. Arch. Pharm..

[138] Sayout A., Ouarhach A., Rabie R., Dilagui I., Soraa N., Romane A. (2020). Evaluation of antibacterial activity of *Lavandulapedunculata* subsp. *atlantica* (Braun-Blanq.) romo essential oil and selected terpenoids against resistant bacteria strains–structure–activity relationships. Biochem. Biodivers..

[139] Wu S.-C., Liu F., Zhu K., Shen J.-Z. (2019). Natural Products That Target Virulence Factors in Antibiotic-Resistant *Staphylococcus aureus*. J. Agric. Food Chem..

[140] Knidel C., Pereira M.F., Barcelos D.H.F., Gomes D.C.d.O., Guimarães M.C.C., Schuenck R.P. (2021). Epigallocatechin gallate has antibacterial and antibiofilm activity in methicillin resistant and susceptible *Staphylococcus aureus* of different lineages in non-cytotoxic concentrations. Nat. Prod. Res..

[141] Shinde S., Lee L.H., Chu T. (2021). Inhibition of Biofilm Formation by the Synergistic Action of EGCG-S and Antibiotics. Antibiotics.

[142] Hengge R. (2019). Targeting Bacterial Biofilms by the Green Tea Polyphenol EGCG. Molecules.

[143] Nunthanawanich P., Sompong W., Sirikwanpong S., Mäkynen K., Adisakwattana S., Dahlan W., Ngamukote S. (2016). *Moringa oleifera* aqueous leaf extract inhibits reducing monosaccharide-induced protein glycation and oxidation of bovine serum albumin. Springerplus.

[144] Menon L., Chouhan O., Walke R., Shah S., Damare S., Biswas S. (2023). Disruption of *Staphylococcus aureus* biofilms with purified *Moringa Oleifera* leaf extract protein. Protein Pept. Lett..

[145] Kim S., Kim T.J. (2024). Inhibitory Effect of *Moringa oleifera* Seed Extract and Its Behenic Acid Component on *Staphylococcus aureus* Biofilm Formation. Antibiotics.

[146] Ali M.K., Galut H.S., Matar E., Mamand S.F., Sabrie L.S., Mala S.F., Jghef M.M. (2025). Exploring the antibacterial and anti-biofilm properties of *Rosmarinus officinalis* extracts: A natural strategy against methicillin-resistant *Staphylococcus aureus*. Open Vet. J..

[147] Iobbi V., Parisi V., Bernabè G., De Tommasi N., Bisio A., Brun P. (2023). Anti-Biofilm Activity of Carnosic Acid from Salvia rosmarinus against Methicillin-Resistant *Staphylococcus aureus*. Plants.

[148] Nakagawa S., Hillebrand G.G., Nunez G. (2020). *Rosmarinus officinalis* L. (Rosemary) Extracts Containing Carnosic Acid and Carnosol are Potent Quorum Sensing Inhibitors of *Staphylococcus aureus* Virulence. Antibiotics.

[149] Khan M.A., Celik I., Khan H.M., Shahid M., Shahzad A., Kumar S., Ahmed B. (2023). Antibiofilm and anti-quorum sensing activity of *Psidium guajava* L. leaf extract: In vitro and in silico approach. PLoS ONE.

[150] Akter F., Ahmed S.M., Jowarder R.J., Nahar S., Razia S., Ferdous J., Afrin M., Manju A.M., Nishat E.F. (2026). Antibacterial Activities of Guava (*Psidium guajava*) Leaf Aqueous Extract Against *Staphylococcus aureus* and *Escherichia coli*. Mymensingh Med. J..

[151] Dutta K., Karmakar A., Jana D., Ballav S., Shityakov S., Panda A.K., Ghosh C. (2020). Benzyl isocyanate isolated from the leaves of *Psidium guajava* inhibits *Staphylococcus aureus* biofilm formation. Biofouling.

[152] Al-Rimawi F., Imtara H., Abbadi J., Sawalha S., Atiya M., Mudalal S., Mauriello G. (2025). Evaluation of antimicrobial efficacy of *Psidium guajava* L. leaf extract in ketoconazole shampoo. Sci. Rep..

[153] Tabti S., Boukraâ D., Itatahine A., Truchado P., Miloudi K., Erenler R., Sami R., Abduljawad S.H., Basri A., Alsulaimani F. (2026). Targeting QS-mediated virulence in *Staphylococcus aureus* using *Eucalyptus globulus* essential oil: From chemistry to molecular docking. Open Chem..

[154] Elangovan S., Mudgil P. (2023). Antibacterial Properties of *Eucalyptus globulus* Essential Oil against MRSA: A Systematic Review. Antibiotics.

[155] Zhang H., Zhang Z., Li J., Qin G. (2023). New Strategies for Biocontrol of Bacterial Toxins and Virulence: Focusing on Quorum-Sensing Interference and Biofilm Inhibition. Toxins.

[156] Okba M.M., El-Shiekh R.A., Abu-Elghait M., Sobeh M., Ashour R.M.S. (2021). HPLC-PDA-ESI-MS/MS profiling and anti-biofilm potential of *Eucalyptussideroxylon* flowers. Antibiotics.

[157] Ashour R.M.S., Okba M.M., Menze E.T., El Gedaily R.A. (2019). *Eucalyptus Sideroxylon* Bark anti-inflammatory potential, its UPLC-PDA-ESI-qTOF-MS profiling, and isolation of a new phloroglucinol. J. Chromatogr. Sci..

[158] Quelemes P.V., Perfeito M.L.G., Guimarães M.A., dos Santos R.C., Lima D.F., Nascimento C., Silva M.P.N., Soares M.J.d.S., Ropke C.D., Eaton P. (2015). Effect of neem (*Azadirachta indica* A. Juss) leaf extract on resistant *Staphylococcus aureus* biofilm formation and *Schistosoma mansoni* worms. J. Ethnopharmacol..

[159] Shil A., Shuvo M.N., Sultana H., Alam N., Himel M.K. (2026). Impact of Neem (*Azadirachta indica*) and Papaya (*Carica papaya*) Leaves on *Staphylococcus aureus* Virulence Factors. Res. Perspect. Biol. Sci..

[160] Ziegert Z., Dietz M., Hill M., McBride M., Painter E., Elias M.H., Staley C. (2024). Targeting quorum sensing for manipulation of commensal microbiota. BMC Biotechnol..

[161] Packiavathy I.A., Priya S., Pandian S.K., Ravi A.V. (2014). Inhibition of biofilm development of uropathogens by curcumin—An anti-quorum sensing agent from *Curcuma longa*. Food Chem..

[162] Adamczak A., Ożarowski M., Karpiński T.M. (2020). Curcumin, a Natural Antimicrobial Agent with Strain-Specific Activity. Pharmaceuticals.

[163] Pu Z., Tang H., Long N., Qiu M., Gao M., Sun F., Dai M. (2020). Assessment of the anti-virulence potential of extracts from four plants used in traditional Chinese medicine against multidrug-resistant pathogens. BMC Complement. Med. Ther..

[164] Silva E., Teixeira J.A., Pereira M.O., Rocha C.M.R., Sousa A.M. (2023). Evolving biofilm inhibition and eradication in clinical settings through plant-based antibiofilm agents. Phytomedicine.

[165] Nair A., Debprasad C., Bhaskar S. (2019). Plant-derived immunomodulators. New Look to Phytomedicine.

[166] Mahmood M.S., Mártinez J.L., Aslam A., Rafique A., Vinet R., Laurido C., Hussain I., Abbas R.Z., Khan A., Ali S. (2016). Antiviral effects of green tea (*Camellia sinensis*) against pathogenic viruses in human and animals (a mini-review). Afr. J. Tradit. Complement. Altern. Med..

[167] Cui Y., Oh Y.J., Lim J., Youn M., Lee I., Pak H.K., Park W., Jo W., Park S. (2012). AFM study of the differential inhibitory effects of the green tea polyphenol (-)-epigallocatechin-3-gallate (EGCG) against Gram-positive and Gram-negative bacteria. Food Microbiol..

[168] Tamfu A.N., Ceylan O., Kucukaydin S., Duru M.E. (2020). HPLC-DAD phenolic profiles, antibiofilm, anti-quorum sensing and enzyme inhibitory potentials of *Camellia sinensis* (L.) O. Kuntze and *Curcuma longa* L. LWT.

[169] Armstrong L., Araújo Vieira do Carmo M., Wu Y., Antônio Esmerino L., Azevedo L., Zhang L., Granato D. (2020). Optimizing the extraction of bioactive compounds from pu-erh tea (*Camellia sinensis* var. assamica) and evaluation of antioxidant, cytotoxic, antimicrobial, antihemolytic, and inhibition of α-amylase and α-glucosidase activities. Food Res. Int..

[170] Yadav N.R., Jain M., Sharma A., Aggarwal A., Pahuja M., Mehta A., Rawal A., Jain V. (2021). Role of a Miracle Tree (*Moringa oleifera*) in Healthcare. J. Evol. Med. Dent. Sci..

[171] de Oliveira A.M., de Abreu Filho B.A., de Jesus Bassetti F., Bergamasco R., Gomes R.G. (2020). Natural extract of moringa oleifera leaves promoting control of *Staphylococcus aureus* strains biofilm on PVC surface. Food Bioprocess Technol..

[172] Enan G., Al-Mohammadi A.-R., Mahgoub S., Abdel-Shafi S., Askar E., Ghaly M.F., Taha M.A., El-Gazzar N. (2020). Inhibition of *Staphylococcus aureus* LC 554891 by *Moringa oleifera* seed extract either singly or in combination with antibiotics. Molecules.

[173] de Oliveira J.R., Camargo S.E.A., de Oliveira L.D. (2019). *Rosmarinus officinalis* L. (rosemary) as therapeutic and prophylactic agent. J. Biomed. Sci..

[174] Jardak M., Elloumi-Mseddi J., Aifa S., Mnif S. (2017). Chemical composition, anti-biofilm activity and potential cytotoxic effect on cancer cells of *Rosmarinus officinalis* L. essential oil from Tunisia. Lipids Health Dis..

[175] de Oliveira J.R., de Jesus D., Figueira L.W., de Oliveira F.E., Pacheco Soares C., Camargo S.E.A., Jorge A.O.C., de Oliveira L.D. (2017). Biological activities of *Rosmarinus officinalis* L. (rosemary) extract as analyzed in microorganisms and cells. Exp. Biol. Med..

[176] Nasr-Eldin M.A., Abdelhamid A., Baraka D. (2017). Antibiofilm and antiviral potential of leaf extracts from *Moringa oleifera* and rosemary (*Rosmarinus officinalis* Lam.). Egypt. J. Microbiol..

[177] Khin M., Knowles S.L., Crandall W.J., Jones D.D., Oberlies N.H., Cech N.B., Houriet J. (2021). Capturing the antimicrobial profile of *Rosmarinus officinalis* against methicillin-resistant *Staphylococcus aureus* (MRSA) with bioassay-guided fractionation and bioinformatics. J. Pharm. Biomed. Anal..

[178] S. L.P., A U., S.J G.F. (2020). Investigation on the biofilm eradication potential of selected medicinal plants against methicillin-resistant *Staphylococcus aureus*. Biotechnol. Rep..

[179] Rasool M., Malik A., Arooj M., Alam M.Z., Alam Q., Awan M., Asif M., Qazi M.H., Zaheer A., Khan S.U. (2017). Evaluation of antimicrobial activity of ethanolic extracts of *Azadirachta indica* and *Psidium guajava* against clinically important bacteria at varying pH and temperature. Biomed. Res.-India.

[180] Qaralleh H., Al-Limoun M., Khlaifat A., Khleifat K., Al-Tawarah N., Alsharafa K., Abu-Harirah H. (2021). Antibacterial and antibiofilm activities of a traditional herbal formula against respiratory infection causing bacteria. arXiv.

[181] Dildar N.D., Ali S.N., Begum S., Andleeb S., SIDDIQUI B.S. (2020). Antibiofilm activity and composition of the petroleum ether soluble fraction of *Psidium guajava* Linn. FUUAST J. Biol..

[182] dos Santos R.M., Costa G., Cerávolo I.P., Dias-Souza M.V. (2020). Antibiofilm potential of *Psidium guajava* and *Passiflora edulis* pulp extracts against *Staphylococcus aureus*, cytotoxicity, and interference on the activity of antimicrobial drugs. Future J. Pharm. Sci..

[183] Penesyan A., Nagy S.S., Kjelleberg S., Gillings M.R., Paulsen I.T. (2019). Rapid microevolution of biofilm cells in response to antibiotics. npj Biofilms Microbiomes.

[184] Mohamed G.A., Ibrahim S.R. (2007). Eucalyptone G, a new phloroglucinol derivative and other constituents from *Eucalyptus Globulus* Labill. Arkivoc.

[185] Brooker M.I.H. *Eucalyptus globulus*. Royal Botanic Gardens Victoria. https://vicflora.rbg.vic.gov.au/flora/taxon/1dfdd922-7fa3-4fc4-bb6e-96cfa112337a.

[186] Mota V.d.S., Turrini R.N., Poveda V.d.B. (2015). Antimicrobial activity of *Eucalyptus globulus* oil, xylitol and papain: A pilot study. Rev. Esc. Enferm. USP.

[187] Merghni A., Noumi E., Hadded O., Dridi N., Panwar H., Ceylan O., Mastouri M., Snoussi M. (2018). Assessment of the antibiofilm and antiquorum sensing activities of *Eucalyptus globulus* essential oil and its main component 1,8-cineole against methicillin-resistant *Staphylococcus aureus* strains. Microb. Pathog..

[188] Kaur S., Sharma N., Aanchal A.G., Sharma A., Sharma A., Sharma V. (2018). Anti-biofilm potential of aqueous Eucalyptus leaf extract against nosocomial pathogens: *Staphylococcus* and *Pseudomonas aeruginosa*. Pharma Innovtion.

[189] Santos B.M.d., Zibrandtsen J.F.S., Gunbilig D., Sørensen M., Cozzi F., Boughton B.A., Heskes A.M., Neilson E.H.J. (2019). Quantification and localization of formylated phloroglucinol compounds (FPCs) in *Eucalyptus* species. Front. Plant Sci..

[190] Mostafa I., Abbas H.A., Ashour M.L., Yasri A., El-Shazly A.M., Wink M., Sobeh M. (2020). Polyphenols from *Salix tetrasperma* impair virulence and inhibit quorum sensing of *Pseudomonas aeruginosa*. Molecules.

[191] Alzohairy M.A. (2016). Therapeutics role of *Azadirachta indica* (Neem) and their active constituents in diseases prevention and treatment. *Evid.-Based Complement*. Altern. Med..

[192] Zihadi M.A.H., Rahman M., Talukder S., Hasan M.M., Nahar S., Sikder M.H. (2019). Antibacterial efficacy of ethanolic extract of *Camellia sinensis* and *Azadirachta indica* leaves on methicillin-resistant *Staphylococcus aureus* and shiga-toxigenic *Escherichia coli*. J. Adv. Vet. Anim. Res..

[193] Sood S., Davis J. (2020). Anti-biofilm activity of *Azadirachta indica* and *Ocimum sanctum* aqueous extract combination against MRSA. Int. J. Pharm. Res..

[194] Omosa L.K., Midiwo J.O., Kuete V. (2017). *Curcuma* *longa*. Medicinal Spices and Vegetables from Africa.

[195] Kamran A., Sadia S. (2017). A comprehensive review on *Curcuma longa* Linn.: Phytochemical, pharmacological, and molecular study. Int. J. Green Pharm..

[196] Tilak J.C., Banerjee M., Mohan H., Devasagayam T.P. (2004). Antioxidant availability of turmeric in relation to its medicinal and culinary uses. Phytother. Res. PTR.

[197] Ribeiro A.P.D., Pavarina A.C., Dovigo L.N., Brunetti I.L., Bagnato V.S., Vergani C.E., de Souza Costa C.A. (2013). Phototoxic effect of curcumin on methicillin-resistant *Staphylococcus aureus* and L929 fibroblasts. Lasers Med. Sci..

[198] Ye Y., Li Y., Fang F. (2014). Upconversion nanoparticles conjugated with curcumin as a photosensitizer to inhibit methicillin-resistant *Staphylococcus aureus* in lung under near infrared light. Int. J. Nanomed..

[199] *Sanguisorba officinalis* L. Plants of the World Online. https://powo.science.kew.org/taxon/urn:lsid:ipni.org:names:741402-1.

[200] Wang S., Luo J., Liu X.-Q., Kang O.-H., Kwon D.-Y. (2021). Antibacterial activity and synergy of antibiotics with sanguisorbigenin isolated from *Sanguisorba officinalis* L. against methicillin-resistant *Staphylococcus aureus*. Lett. Appl. Microbiol..

[201] Zhu H.-l., Chen G., Chen S.-n., Wang Q.-r., Wan L., Jian S.-p. (2019). Characterization of polyphenolic constituents from *Sanguisorba officinalis* L. and its antibacterial activity. Eur. Food Res. Technol..

[202] Jang Y.S., Mosolygó T. (2020). Inhibition of bacterial biofilm formation by phytotherapeutics with focus on overcoming antimicrobial resistance. Curr. Pharm. Des..

[203] Chen X., Shang F., Meng Y., Li L., Cui Y., Zhang M., Qi K., Xue T. (2015). Ethanol extract of *Sanguisorba officinalis* L. inhibits biofilm formation of methicillin-resistant *Staphylococcus aureus* in an ica-dependent manner. J. Dairy Sci..

[204] Jang E., Inn K.-S., Jang Y.P., Lee K.-T., Lee J.-H. (2018). Phytotherapeutic activities of *Sanguisorba officinalis* and its chemical constituents: A Review. Am. J. Chin. Med..

[205] Manilal A., Sabu K.R., Shewangizaw M., Aklilu A., Seid M., Merdekios B., Tsegaye B. (2020). In vitro antibacterial activity of medicinal plants against biofilm-forming methicillin-resistant *Staphylococcus aureus*: Efficacy of *Moringa stenopetala* and *Rosmarinus officinalis* extracts. Heliyon.

[206] Mohamed E.H., Abdel-Hafez S.M., Soliman M.M., Alotaibi S.H., Alkhedaide A., Mostafa M.A. (2020). Characterization and identification of methicillin-resistant *Staphylococcus aureus* (MRSA) producing biofilm: Impacts of garlic extract and *Lactobacillus* biosurfactants. Biomed. Pharmacol. J..

[207] Tamfu A.N., Ceylan O., Fru G.C., Ozturk M., Duru M.E., Shaheen F. (2020). Antibiofilm, antiquorum sensing and antioxidant activity of secondary metabolites from seeds of *Annona senegalensis*, Persoon. Microb. Pathog..

[208] Utami R., Aini S.R., Wirasisya D.G. (2019). The Antibiofilm Activity of *Jatropha curcas* L. Latex on *Staphylococcus aureus* ATCC 25923. Nat. B J. Health Environ. Sci..

[209] Kim J.-H., Han S.-Y., Kwon J.-H., Lee D.-S. (2020). *Orostachys japonicus* ethyl acetate fraction suppresses MRSA biofilm formation. Asian Pac. J. Trop. Med..

[210] Hadis M., Gebreyohannes Y., Gemeda N. (2020). Potential therapeutic uses of *Moringa stenopetala*: A scoping review. J. Glob. Health Sci..

[211] Uc-Cachón A.H., Dzul-Beh A.d.J., Palma-Pech G.A., Jiménez-Delgadillo B., Flores-Guido J.S., Gracida-Osorno C., Molina-Salinas G.M. (2021). Antibacterial and antibiofilm activities of Mayan medicinal plants against methicillin-susceptible and -resistant strains of *Staphylococcus aureus*. J. Ethnopharmacol..

[212] Acquaviva R., D’Angeli F., Malfa G.A., Ronsisvalle S., Garozzo A., Stivala A., Ragusa S., Nicolosi D., Salmeri M., Genovese C. (2021). Antibacterial and anti-biofilm activities of walnut pellicle extract (*Juglans regia* L.) against coagulase-negative staphylococci. Nat. Prod. Res..

[213] M Z. (2020). Antimicrobial and anti-biofilm activities of *Citrus sinensis* and *Moringa oleifera* against the pathogenic *Pseudomonas aeruginosa* and *Staphylococcus aureus*. Cureus.

[214] Parai D., Islam E., Mitra J., Mukherjee S.K. (2017). Effect of Bacoside A on growth and biofilm formation by *Staphylococcus aureus* and *Pseudomonas aeruginosa*. Can. J. Microbiol..

[215] Diniz R.C., Soares L.W., Nascimento da Silva L.C. (2018). Virtual screening for the development of new effective compounds against *Staphylococcus aureus*. Curr. Med. Chem..

[216] Huda N., Dwiyanti R.D., Thuraidah A. (2019). Effectiveness of Cinnamon (*Cinnamomum burmannii*) ethanol extract against *Staphylococcus aureus* growth. Trop. Health Med. Res..

[217] Wijesinghe G.K., Feiria S.B., Maia F.C., Oliveira T.R., Joia F., Barbosa J.P., Boni G.C., HÖfling J.F. (2021). In-vitro antibacterial and antibiofilm activity of *Cinnamomum verum* leaf oil against *Pseudomonas aeruginosa*, *Staphylococcus aureus* and *Klebsiella pneumoniae*. An. DA Acad. Bras. DE Ciênc..

[218] Nuță D.C., Limban C., Chiriță C., Chifiriuc M.C., Costea T., Ioniță P., Nicolau I., Zarafu I. (2021). Contribution of essential oils to the fight against microbial biofilms—A review. Processes.

[219] Bazargani M.M., Rohloff J. (2016). Antibiofilm activity of essential oils and plant extracts against *Staphylococcus aureus* and *Escherichia coli* biofilms. Food Control.

[220] Alexa E., Danciu C., Radulov I., Obistioiu D., Sumalan R.M., Morar A., Dehelean C.A. (2018). Phytochemical screening and biological activity of *Mentha* × *piperita* L. and *Lavandula angustifolia* Mill. extracts. Anal. Cell. Pathol..

[221] Oral N.B., Vatansever L., Aydin B.D., Sezer C., Guven A., Gumez M., Kurkcuoglu M. (2010). Effect of oregano essential oil on biofilms formed by *Staphylococci* and *Escherichia coli*. Kafkas Univ. Vet. Fak. Derg..

[222] Quave C.L., Plano L.R.W., Pantuso T., Bennett B.C. (2008). Effects of extracts from Italian medicinal plants on planktonic growth, biofilm formation and adherence of methicillin-resistant *Staphylococcus aureus*. J. Ethnopharmacol..

[223] Przerwa F., Kukowka A., Uzar I. (2020). *Ballota nigra* L.—An overview of pharmacological effects and traditional uses. Int. J..

[224] Mordmuang A., Brouillette E., Voravuthikunchai S.P., Malouin F. (2019). Evaluation of a *Rhodomyrtus tomentosa* ethanolic extract for its therapeutic potential on *Staphylococcus aureus* infections using in vitro and in vivo models of mastitis. Vet. Res..

[225] Mohd Amin I., Mohamad Zain N., Ahmad V.N., Mat Daud D.N., Abdullah Sani N. (2019). Antimicrobial and antibiofilm activities of the methanol extracts of *Rosa damascena* against *S. aureus*. Proceedings of the 9th Dental Student’s Scientific Symposium.

[226] Hemmati F., Salehi R., Ghotaslou R., Kafil H.S., Hasani A., Gholizadeh P., Rezaee M.A. (2020). The assessment of antibiofilm activity of chitosan-zinc oxide-gentamicin nanocomposite on *Pseudomonas aeruginosa* and *Staphylococcus aureus*. Int. J. Biol. Macromol..

[227] Quave C.L., Plano L.R., Bennett B.C. (2011). Quorum sensing inhibitors of *Staphylococcus aureus* from Italian medicinal plants. Planta Medica.

[228] Mulat M., Khan F., Pandita A. (2021). Biochemical composition, antibacterial and anti-biofilm activities of Indian medicinal plants. Anti-Infect. Agents.

[229] Utami P.S.M., Noorhamdani N., Rahayu M. (2020). The extract of kemangi leaves as inhibitor of biofilm from *Staphylococcus aureus* in vitro. J. Agromedicine Med. Sci..

[230] Mouwakeh A., Kincses A., Nové M., Mosolygó T., Mohácsi-Farkas C., Kiskó G., Spengler G. (2019). *Nigella sativa* essential oil and its bioactive compounds as resistance modifiers against *Staphylococcus aureus*. Phytother. Res..

[231] Gawron G., Krzyczkowski W., Lemke K., Ołdak A., Kadziński L., Banecki B. (2019). *Nigella sativa* seed extract applicability in preparations against methicillin-resistant *Staphylococcus aureus* and effects on human dermal fibroblasts viability. J. Ethnopharmacol..

[232] Gómez-Sequeda N., Cáceres M., Stashenko E.E., Hidalgo W., Ortiz C. (2020). Antimicrobial and antibiofilm activities of essential oils against *Escherichia coli* O157: H7 and methicillin-resistant *Staphylococcus aureus* (MRSA). Antibiotics.

[233] Kryvtsova M., Salamon I., Koscova J., Bucko D., Spivak M. (2019). Antimicrobial, antibiofilm and biochemichal properties of *Thymus vulgaris* essential oil against clinical isolates of opportunistic infections. Biosyst. Divers..

[234] de Oliveira J.R., Figueira L.W., Sper F.L., Meccatti V.M., Camargo S.E.A., de Oliveira L.D. (2017). *Thymus vulgaris* L. and thymol assist murine macrophages (RAW 264.7) in the control of in vitro infections by *Staphylococcus aureus*, *Pseudomonas aeruginosa*, and *Candida albicans*. Immunol. Res..

[235] Cech N.B., Junio H.A., Ackermann L.W., Kavanaugh J.S., Horswill A.R. (2012). Quorum quenching and antimicrobial activity of goldenseal (*Hydrastis canadensis*) against methicillin-resistant *Staphylococcus aureus* (MRSA). Planta Medica.

[236] Pervaiz A., Khan R., Anwar F., Mushtaq G., A Kamal M., Khan H. (2016). Alkaloids: An emerging antibacterial modality against methicillin resistant *Staphylococcus aureus*. Curr. Pharm. Des..

[237] Chemsa A.E., Erol E., Öztürk M., Zellagui A., Özgür C., Gherraf N., Duru M.E. (2016). Chemical constituents of essential oil of endemic *Rhanterium suaveolens* Desf. growing in Algerian Sahara with antibiofilm, antioxidant and anticholinesterase activities. Nat. Prod. Res..

[238] Ben Abdallah F., Lagha R., Gaber A. (2020). Biofilm inhibition and eradication properties of medicinal plant essential oils against methicillin-resistant *Staphylococcus aureus* clinical isolates. Pharmaceuticals.

[239] Endo E.H., Costa G.M., Makimori R.Y., Ueda-Nakamura T., Nakamura C.V., Dias Filho B.P. (2018). Anti-biofilm activity of *Rosmarinus officinalis*, *Punica granatum* and *Tetradenia riparia* against methicillin-resistant *Staphylococcus aureus* (MRSA) and synergic interaction with penicillin. J. Herb. Med..

[240] Kodi M.K., Hassan M.H., Nader M.I. (2018). Effect of alcoholic extracts of *Rosmarinus officinalis* and *Propolis* on inhibition of *Staphylococcus aureus* and *Klebsiella pneumoniae* biofilm isolate from urinary tract infection. J. Univ. Anbar Pure Sci..

[241] Craft K.M., Nguyen J.M., Berg L.J., Townsend S.D. (2019). Methicillin-resistant *Staphylococcus aureus* (MRSA): Antibiotic-resistance and the biofilm phenotype. MedChemComm.

[242] Quave C.L., Estévez-Carmona M., Compadre C.M., Hobby G., Hendrickson H., Beenken K.E., Smeltzer M.S. (2012). Ellagic acid derivatives from Rubus ulmifolius inhibit *Staphylococcus aureus* biofilm formation and improve response to antibiotics. PLoS ONE.

[243] Chung P.Y., Toh Y.S. (2014). Anti-biofilm agents: Recent breakthrough against multi-drug resistant *Staphylococcus aureus*. Pathog. Dis..

[244] Kou J., Xin T.Y., McCarron P., Gupta G., Dureja H., Satija S., Mehta M., Bakshi H.A., Tambuwala M.M., Collet T. (2020). Going beyond antibiotics: Natural plant extracts as an emergent strategy to combat biofilm-associated infections. J. Environ. Pathol. Toxicol. Oncol..

[245] Kačániová M., Galovičová L., Ivanišová E., Vukovic N.L., Štefániková J., Valková V., Borotová P., Žiarovská J., Terentjeva M., Felšöciová S. (2020). Antioxidant, antimicrobial and antibiofilm activity of coriander (*Coriandrum sativum* L.) essential oil for its application in foods. Foods.

[246] Yincharoen K., Adekoya A.E., Chokpaisarn J., Kunworarath N., Jaisamut P., Limsuwan S., Chusri S. (2021). Anti-infective effects of traditional household remedies described in the national list of essential medicines, Thailand, on important human pathogens. J. Herb. Med..

[247] Khan M.F., Tang H., Lyles J.T., Pineau R., Mashwani Z.-u.-R., Quave C.L. (2018). Antibacterial properties of medicinal plants from Pakistan against multidrug-resistant ESKAPE pathogens. Front. Pharmacol..

[248] Hassan H.A., Abdelwahab S.F., Desoukey S.Y., Mohamed K.M., Kamel M.S. (2019). Comparative study of antimicrobial activity of seven ficus species cultivated in Egypt. Indian J. Public Health Res. Dev..

[249] Awolola G.V., Koorbanally N.A., Chenia H., Shode F.O., Baijnath H. (2014). Antibacterial and anti-biofilm activity of flavonoids and triterpenes isolated from the extracts of *Ficus sansibarica* Warb. subsp. *sansibarica* (*Moraceae*) extracts. Afr. J. Tradit. Complement. Altern. Med..

[250] Haq A., Siddiqi M., Batool S.Z., Islam A., Khan A., Khan D., Khan S., Khan H., Shah A.A., Hasan F. (2019). Comprehensive investigation on the synergistic antibacterial activities of *Jatropha curcas* pressed cake and seed oil in combination with antibiotics. AMB Express.

[251] Chusri S., Sinvaraphan N., Chaipak P., Luxsananuwong A., Voravuthikunchai S.P. (2014). Evaluation of antibacterial activity, phytochemical constituents, and cytotoxicity effects of Thai household ancient remedies. J. Altern. Complement. Med..

[252] Triveni A., Kumar M.S., T Shivannavar C., Gaddad S.M. (2016). Antibacterial and antibiofilm activities of crude extracts of *Lawsonia inermis* against methicillin-resistant *Staphylococcus aureus*. Asian J. Pharm. Clin. Res..

[253] Edziri H., Jaziri R., Chehab H., Verschaeve L., Flamini G., Boujnah D., Hammami M., Aouni M., Mastouri M. (2019). A comparative study on chemical composition, antibiofilm and biological activities of leaves extracts of four *Tunisian olive* cultivars. Heliyon.

[254] Sidkey B.A., Omran A.H. (2017). Evaluation of the antibacterial effects of *Eucalyptus camaldulensis* L., *Glycyrrhiza glabra* L. and *Morus nigra* L. extracts against some pathogenic bacteria in vitro. Iraqi J. Sci..

[255] Subramanian R., Chandra M., Yogapriya S., Aravindh S., Ponmurugan K. (2016). Isolation of methyl gallate from mango twigs and its anti-biofilm activity. J. Biol. Act. Prod. Nat..

[256] Al-Gbouri N., Hamzah A. (2018). Evaluation of *Phyllanthus emblica* extract as antibacterial and antibiofilm against biofilm formation bacteria. Iraqi J. Agric. Sci..

[257] Liu T., Wang J., Gong X., Wu X., Liu L., Chi F. (2020). Rosemary and tea tree essential oils exert antibiofilm activities in vitro against *Staphylococcus aureus* and *Escherichia coli*. J. Food Prot..

[258] Zhao X., Liu Z., Liu Z., Meng R., Shi C., Chen X., Bu X., Guo N. (2018). Phenotype and RNA-seq-based transcriptome profiling of *Staphylococcus aureus* biofilms in response to tea tree oil. Microb. Pathog..

[259] Li K., Han X., Li R., Xu Z., Pan T., Liu J., Li B., Wang S., Diao Y., Liu X. (2019). Composition, antivirulence activity, and active property distribution of the fruit of *Terminalia chebula* Retz. J. Food Sci..

[260] Ouyang P., He X., Yuan Z.-W., Yin Z.-Q., Fu H., Lin J., He C., Liang X., Lv C., Shu G. (2018). Erianin against *Staphylococcus aureus* infection via inhibiting *sortase A*. Toxins.

[261] Anupam R., Bagde S., Mondal R. (2020). Antibiofilm and synergistic antibacterial effect of Garlic (*Allium sativum*) against Methicillin resistant *Staphylococcus aureus* (MRSA). Int. J. Infect. Dis..

[262] Zhang H., Wang J. (2019). Constituents of the essential oils of garlic and citronella and their vapor-phase inhibition mechanism against *S. aureus*. Food Sci. Technol. Res..

[263] Morán A., Gutiérrez S., Martínez-Blanco H., Ferrero M., Monteagudo-Mera A., Rodríguez-Aparicio L. (2014). Non-toxic plant metabolites regulate Staphylococcus viability and biofilm formation: A natural therapeutic strategy useful in the treatment and prevention of skin infections. Biofouling.

[264] Zhang Y., Wang J.F., Dong J., Wei J.Y., Wang Y.N., Dai X.H., Wang X., Luo M.J., Tan W., Deng X.M. (2013). Inhibition of α-toxin production by subinhibitory concentrations of naringenin controls *Staphylococcus aureus* pneumonia. Fitoterapia.

[265] Valliammai A., Sethupathy S., Ananthi S., Priya A., Selvaraj A., Nivetha V., Aravindraja C., Mahalingam S., Pandian S.K. (2020). Proteomic profiling unveils citral modulating expression of IsaA, CodY and SaeS to inhibit biofilm and virulence in methicillin-resistant *Staphylococcus aureus*. Int. J. Biol. Macromol..

[266] Fanin M., Fanin É.L.B.B., dos Santos I.C., de Lima J.S., Gonçalves A.P.P., de Almeida Martins L.J.M.V. (2020). Métodos alternativos no tratamento de infecções causadas por *Staphylococcus aureus*. Med. Veterinária.

[267] Seshadri V.D., Vijayaraghavan P., Kim Y.-O., Kim H.-J., Al-Ghamdi A.A., Elshikh M.S., Al-Dosary M.A., Alsubaie Q.D. (2020). In vitro antioxidant and cytotoxic activities of polyherbal extracts from *Vetiveria zizanioides*, *Trichosanthes cucumerina,* and *Mollugo cerviana* on HeLa and MCF-7 cell lines. Saudi J. Biol. Sci..

[268] Kannappan A., Gowrishankar S., Srinivasan R., Pandian S.K., Ravi A.V. (2017). Antibiofilm activity of *Vetiveria zizanioides* root extract against methicillin-resistant *Staphylococcus aureus*. Microb. Pathog..

[269] Song X., Liu T., Wang L., Liu L., Li X., Wu X. (2020). Antibacterial effects and mechanism of mandarin (*Citrus reticulata* L.) essential oil against *Staphylococcus aureus*. Molecules.

[270] Junren C., Xiaofang X., Mengting L., Qiuyun X., Gangmin L., Huiqiong Z., Guanru C., Xin X., Yanpeng Y., Fu P. (2021). Pharmacological activities and mechanisms of action of *Pogostemon cablin* Benth: A review. Chin. Med..

[271] Shahid A., Rasool M., Akhter N., Aslam B., Hassan A., Sana S., Rasool M.H., Khurshid M. (2019). Innovative strategies for the control of biofilm formation in clinical settings. Bacterial Biofilms.

[272] Nadaf N.H., Parulekar R.S., Patil R.S., Gade T.K., Momin A.A., Waghmare S.R., Dhanavade M.J., Arvindekar A.U., Sonawane K.D. (2018). Biofilm inhibition mechanism from extract of *Hymenocallis littoralis* leaves. J. Ethnopharmacol..

[273] Gao S., Liu G., Li J., Chen J., Li L., Li Z., Zhang X., Zhang S., Thorne R.F., Zhang S. (2020). Antimicrobial Activity of Lemongrass Essential Oil (*Cymbopogon flexuosus*) and Its Active Component Citral Against Dual-Species Biofilms of *Staphylococcus aureus* and Candida Species. Front. Cell. Infect. Microbiol..

[274] da Silva Gündel S., de Souza M.E., Quatrin P.M., Klein B., Wagner R., Gündel A., de Almeida Vaucher R., Santos R.C.V., Ourique A.F. (2018). Nanoemulsions containing *Cymbopogon flexuosus* essential oil: Development, characterization, stability study and evaluation of antimicrobial and antibiofilm activities. Microb. Pathog..

[275] Zheng X., Chen L., Zeng W., Liao W., Wang Z., Tian X., Fang R., Sun Y., Zhou T. (2021). Antibacterial and anti-biofilm efficacy of Chinese Dragon’s Blood against *Staphylococcus aureus* isolated from infected wounds. Front. Microbiol..

[276] Thu Z.M., Myo K.K., Aung H.T., Armijos C., Vidari G. (2020). Flavonoids and stilbenoids of the genera *Dracaena* and *Sansevieria*: Structures and bioactivities. Molecules.

[277] Akhter F., Islam Q.S., Adib M., Al Faruq A., Ibrahim M. (2019). Phytochemical screenings and bioactivities of *Duabanga grandiflora* (Roxb. ex DC.) Walp. Bangladesh Pharm. J..

[278] Santiago C., Lim K.-H., Loh H.-S., Ting K.N. (2015). Inhibitory effect of *Duabanga grandiflora* on MRSA biofilm formation via prevention of cell-surface attachment and PBP2a production. Molecules.

[279] Cunha B.G., Duque C., Caiaffa K.S., Massunari L., Catanoze I.A., dos Santos D.M., de Oliveira S.H.P., Guiotti A.M. (2020). Cytotoxicity and antimicrobial effects of citronella oil (*Cymbopogon nardus*) and commercial mouthwashes on *S. aureus* and *C. albicans* biofilms in prosthetic materials. Arch. Oral Biol..

[280] Hidayah A.N., Wasito E.B., Debora K., Basori A., Isnaeni I., Utomo B. (2019). Correlation between the bacteriostatic and bactericide effect with antibiofilm and anticolony spreading from javanese citronella oil on methicillin-resistant *Staphylococcus aureus* (MRSA). Folia Medica Indones..

[281] Askarinia M., Ganji A., Jadidi-Niaragh F., Hasanzadeh S., Mohammadi B., Ghalamfarsa F., Ghalamfarsa G., Mahmoudi H. (2019). A review on medicinal plant extracts and their active ingredients against methicillin-resistant and methicillin-sensitive *Staphylococcus aureus*. J. Herbmed Pharmacol..

[282] Chimnoi N., Reuk-Ngam N., Chuysinuan P., Khlaychan P., Khunnawutmanotham N., Chokchaichamnankit D., Thamniyom W., Klayraung S., Mahidol C., Techasakul S. (2018). Characterization of essential oil from *Ocimum gratissimum* leaves: Antibacterial and mode of action against selected gastroenteritis pathogens. Microb. Pathog..

[283] Melo R.S., Albuquerque Azevedo Á M., Gomes Pereira A.M., Rocha R.R., Bastos Cavalcante R.M., Carneiro Matos M.N., Ribeiro Lopes P.H., Gomes G.A., Soares Rodrigues T.H., Santos H.S.D. (2019). Chemical composition and antimicrobial effectiveness of *Ocimum gratissimum* L. essential oil against multidrug-resistant isolates of *Staphylococcus aureus* and *Escherichia coli*. Molecules.

[284] Zhou K., Shi M., Chen R., Zhang Y., Sheng Y., Tong C., Cao G., Shou D. (2025). Natural phytochemical-based strategies for antibiofilm applications. Chin. Med..

[285] Vaou N., Stavropoulou E., Voidarou C., Tsigalou C., Bezirtzoglou E. (2021). Towards Advances in Medicinal Plant Antimicrobial Activity: A Review Study on Challenges and Future Perspectives. Microorganisms.

[286] Aljabali A.A.A., Obeid M.A., Bashatwah R.M., Qnais E., Gammoh O., Alqudah A., Mishra V., Mishra Y., Khan M.A., Parvez S. (2025). Phytochemicals in Cancer Therapy: A Structured Review of Mechanisms, Challenges, and Progress in Personalized Treatment. Chem. Biodivers..

[287] El-Saadony M.T.T., Saad A.M., Mohammed D.M., Korma S.A., Alshahrani M.Y., Ahmed A.E., Ibrahim E.H., Salem H.M., Alkafaas S.S., Saif A.M. (2025). Medicinal plants: Bioactive compounds, biological activities, combating multidrug-resistant microorganisms, and human health benefits—A comprehensive review. Front. Immunol..

[288] Touati A., Mairi A., Ibrahim N.A., Idres T. (2025). Essential Oils for Biofilm Control: Mechanisms, Synergies, and Translational Challenges in the Era of Antimicrobial Resistance. Antibiotics.

[289] Idakwoji P.A., Tukur U., Onwukwe B.O., Agboola J.B., Onugwu E.O., Iyiola A.T., Amadi B.E., Onoja A.O., Ali A.S., Ojo S.S. (2026). Phytochemicals as promising lead compounds for drug discovery: A comprehensive review of mechanisms and pharmacological potentials. Lett. Drug Des. Discov..

